# Cortical Regions Encoding Hardness Perception Modulated by Visual Information Identified by Functional Magnetic Resonance Imaging With Multivoxel Pattern Analysis

**DOI:** 10.3389/fnsys.2019.00052

**Published:** 2019-10-01

**Authors:** Yuri Kim, Nobuo Usui, Atsushi Miyazaki, Tomoki Haji, Kenji Matsumoto, Masato Taira, Katsuki Nakamura, Narumi Katsuyama

**Affiliations:** ^1^Primate Research Institute, Kyoto University, Inuyama, Japan; ^2^Department of Cognitive Neurobiology, Tokyo Medical and Dental University, Tokyo, Japan; ^3^Center for Brain Integration Research, Tokyo Medical and Dental University, Tokyo, Japan; ^4^Tamagawa University Brain Science Institute, Tokyo, Japan

**Keywords:** somatic sensation, multimodal integration, active touch, mirror visual feedback, parietal operculum, intraparietal sulcus, extrastriate body area

## Abstract

Recent studies have revealed that hardness perception is determined by visual information along with the haptic input. This study investigated the cortical regions involved in hardness perception modulated by visual information using functional magnetic resonance imaging (fMRI) and multivoxel pattern analysis (MVPA). Twenty-two healthy participants were enrolled. They were required to place their left and right hands at the front and back, respectively, of a mirror attached to a platform placed above them while lying in a magnetic resonance scanner. In conditions SFT, MED, and HRD, one of three polyurethane foam pads of varying hardness (soft, medium, and hard, respectively) was presented to the left hand in a given trial, while only the medium pad was presented to the right hand in all trials. MED was defined as the control condition, because the visual and haptic information was congruent. During the scan, the participants were required to push the pad with the both hands while observing the reflection of the left hand and estimate the hardness of the pad perceived by the right (hidden) hand based on magnitude estimation. Behavioral results showed that the perceived hardness was significantly biased toward softer or harder in >73% of the trials in conditions SFT and HRD; we designated these trials as visually modulated (SFTvm and HRDvm, respectively). The accuracy map was calculated individually for each of the pair-wise comparisons of (SFTvm vs. MED), (HRDvm vs. MED), and (SFTvm vs. HRDvm) by a searchlight MVPA, and the cortical regions encoding the perceived hardness with visual modulation were identified by conjunction of the three accuracy maps in group analysis. The cluster was observed in the right sensory motor cortex, left anterior intraparietal sulcus (aIPS), bilateral parietal operculum (PO), and occipito-temporal cortex (OTC). Together with previous findings on such cortical regions, we conclude that the visual information of finger movements processed in the OTC may be integrated with haptic input in the left aIPS, and the subjective hardness perceived by the right hand with visual modulation may be processed in the cortical network between the left PO and aIPS.

## Introduction

Tactile texture perception provides essential information for not only recognition of objects (Klatzky et al., 1987) but also manipulation of objects (Johansson and Flanagan, 2009). Hardness is one of the fundamental dimensions that determine the perception of the tactile texture properties, along with roughness, stickiness, and temperature (Okamoto et al., 2013; Bensmaia, 2016). Previous studies have revealed that when one actively touches a deformable object with the hand, the compressional force to the fingers provides important information regarding the perceived hardness (softness) of the objects (Srinivasan and LaMotte, 1995; Friedman et al., 2008). This information is likely conveyed by the activity of slowly adapting type 1 (SA1) fibers, which innervate mechanoreceptors in superficial layers of the skin and respond to steady skin indentation with a sustained discharge (Srinivasan and LaMotte, 1996). Thus, it is clear that hardness perception is determined primarily by haptic signals from the periphery.

However, recent studies have revealed that hardness perception by active touch is also affected by visual information along with haptic inputs. Using virtual reality techniques, Kuschel et al. (2010) and Cellini et al. (2013) investigated the effect of vision on hardness (softness) perception. In these studies, visual information was presented via movies created by computer graphics, in which a deformable surface was pushed by a non-corporeal object, such as a ball or cylinder. Haptic information was provided to participants by the squeezing of two levers with simulated repulsive force (Kuschel et al., 2010) or by the touching of silicone specimens with the hand (Cellini et al., 2013). The indentation of the virtual surface on the display was controlled such that it was congruent or incongruent with that simulated by the haptic deformation. The results indicated that the perceived hardness in the incongruent condition was biased toward harder or softer when the indentation of the virtual surface on the display was smaller or larger, respectively. Furthermore, Punpongsanon et al. (2015) showed that the perceived softness is enhanced when participants push an object with their hand while observing an augmented (more indented) surface induced by a projection mapping technique. Together, these studies demonstrate how incongruent visual information can distort our perception of hardness.

These studies employed virtual reality techniques to experimentally manipulate the visual and haptic stimuli. In our previous study (Katsuyama et al., 2018), we attempted to examine the effect of visual information on hardness perception by active touch under a more natural condition. For this purpose, we used a mirror visual feedback (MVF) paradigm, in which the reflection of the hand appears to be the hand hidden behind the mirror. Participants were required to touch a polyurethane foam pad with both hands placed at the front and back of a mirror at the midline. To the hand at the front side of the mirror, one pad of three different levels of hardness (hard, medium, and soft) was presented in a given trial, while only the medium pad was presented to the hidden hand in all trials. The participants were required to estimate the hardness of the pad perceived by the hidden hand while observing the reflection. The result indicated that the perceived hardness assessed by magnitude estimation was significantly larger or smaller when participants observed the reflection of the hand pushing the harder or softer pad, respectively, at the same time as the hidden hand was pushing a pad of constant hardness. The modulation of the perceived hardness disappeared when participants touched the pads with both hands at different times or with their eyes closed, indicating that the visual modulation was not induced by the interaction of the motor commands for or haptic inputs from both hands in the brain. Further examination revealed that most participants utilized the visual cues regarding both the hand and the pad, such as the finger displacement and indentation of the surface of the pad, to infer the hardness. These results indicate that the modulation of hardness perception is induced by observation of the reflection of a hand pushing a pad of different hardness at the same time as the hidden hand was presented with a pad of constant hardness. Collectively, these findings indicate that hardness perception by active touch is affected by visual information along with haptic inputs.

This study aimed to investigate the cortical regions involved in hardness perception by active touch induced by multimodal integration. For this purpose, we conducted a functional magnetic resonance imaging (fMRI) experiment with a whole-brain searchlight multivoxel pattern analysis (MVPA) along with a conventional univariate analysis. However, it is difficult to distinguish the cortical activity involved in the perception of hardness from that involved in the motor commands for the finger movements. When one touches a deformable object with the hand, the compressional force to the fingers varies with the finger movements, particularly the finger displacement into the object. Since perceived hardness (softness) relies on the peak compressional force to the fingers (Srinivasan and LaMotte, 1995; Friedman et al., 2008), it is plausible that the cortical activity related to hardness perception and motor commands for finger movements may also co-vary, which allows them to be indistinguishable. This is particularly valid when applying the conventional subtraction technique and correlation analysis because these methods are based on the assumption that the intensity of cortical activity differs among cognitive conditions (Petersen et al., 1989; Friston et al., 1996; Amaro and Barker, 2006). To overcome this problem, we employed the hardness estimation task under the MVF that we used in our previous study. Our previous study showed that finger displacement of the hidden hand was constant across all conditions, which suggests that cortical activity related to the motor command and haptic input from the hidden hand should be constant as well while those involved in the perceived hardness cannot. This may allow us to successfully separate the cortical regions specific to the perceived hardness from those specific to the motor command.

Many studies have demonstrated that tactile illusions, including the rubber hand illusion, referred sensation, and MVF, are accompanied by illusory ownership toward the fake hands (Botvinick and Cohen, 1998; Ehrsson et al., 2004; Tsakiris et al., 2006; Bertamini et al., 2011; Bekrater-Bodmann et al., 2014). In particular, when the dummy and hidden hands are moving simultaneously, participants also experience the sensation that the dummy hand is moved by their own will; this is known as the sense of agency (Sanchez-Vives et al., 2010; Kalckert and Ehrsson, 2012; Jenkinson and Preston, 2015; Katsuyama et al., 2018). Therefore, we also investigated whether participants experienced the sense of hand ownership of the image of the hand in the mirror during the hardness estimation task with MVF.

## Materials and Methods

### Participants

Twenty-two healthy individuals (mean age 21.0 ± 0.64 years, eight females) participated in this study. They were all right-handed (handedness score = 89.6 ± 11.8, measured with the Edinburgh Handedness Inventory, Oldfield, 1971). They had normal or corrected to-normal vision. All participants provided written informed consent before the experiment. This study was approved by the local Ethical Committee for the Faculty of Dentistry of the Tokyo Medical and Dental University (D2016-051) Primate Research Institute of Kyoto University (2016-13), and Tamagawa University (N27-42), and corresponded to the Human Subjects Guidelines of the Declaration of Helsinki.

### Apparatus

Three polyurethane foam pads (9 × 7 × 4 cm, length × width × height) of varying degrees of hardness were used (Inoac Corporation, Nagoya, Japan). They were labeled S (soft), M (medium), and H (hard) in increasing order of hardness. The physical hardness of each pad was calculated on the basis of the elastic modulus (S: 1871.7, M: 3434.6, H: 9803.9 N/cm^2^). They were wrapped in a green cloth such that the participants were unable to judge the hardness of the pads based on the appearance.

**FIGURE 1:**
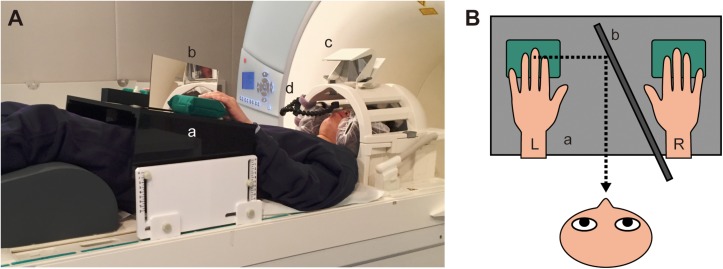
Set up of the functional magnetic resonance imaging experiment. **(A)** Participants were required to lie on the scanner table in the supine position with both hands placed on the platform **(a)**. The left and right hands were placed at the front and back of a mirror, respectively **(b)**. The participants could observe the reflection of the left hand via another mirror on the top of head coil **(c)**. The oral responses of participants were recorded by a microphone attached to the head coil **(d)**. Visual cues were presented on a monitor placed at the other side of the room (not shown), and participants could see the cues reflected in the mirror **(c)**. **(B)** A schematic diagram showing the top view of the platform during the scan. a: top table of the platform, b: mirror on the platform, L: left hand, R: right hand. Participants could observe the reflection of the left hand through another mirror on the head coil (not shown), whereas the right hand was hidden behind the mirror on the platform.

To perform MVF lying in the scanner, an acrylic platform (Figures 1A,Ba) with a mirror (Figures 1A,Bb) on the top table was prepared. The platform was placed over the body of the participants while they were lying in the MR scanner. The left and right hands were placed at the front and back of the mirror, respectively. They could observe the reflection of the left hand on the platform in another mirror (Figure 1Ac) attached on the top of the head coil, while the right hand was hidden behind the mirror on the platform (Figure 1B). The position, height, and angle of the mirror on the platform were adjusted individually such that the reflection of the left hand appeared to be the right hand. The position of both hands was also adjusted at the beginning of every run. Visual cues were presented on a flat monitor placed 5 m from the entrance of the bore of the scanner. Participants could observe the cues in the mirror on the head coil. The horizontal and vertical visual angle of the mirror was 36.2 and 25.0 degrees, respectively. All participants could observe the visual cues in their entirety and the mirror reflection of the left hand on the platform through the mirror. The oral responses of participants were recorded by a voice-capture system for MRI experiments (FOMRI^TM^ II, Optoacoustics Ltd., Or Yehuda, Israel) via a microphone attached to the head coil (Figure 1Ad).

### Hardness Estimation Task

#### Pilot Study

We conducted a pilot study before the main experiment, to confirm whether the visual modulation of hardness perception can be induced in a supine position in the MR scanner (see Supplementary Material for details of the study). All apparatus, including the scanner, pads, and platform, were the same as those used in the main experiment. The participants performed the hardness estimation task while lying in the scanner. The procedure for the task was basically the same as that in the main experiment described below. Along with the three pad conditions (SFT, MED, and HRD), two timing conditions (SYNC and ASYNC) were employed. Participants touched the pad with both hands synchronously and asynchronously in the SYNC and ASYNC conditions, respectively, while observing the mirror reflection of the left hand. The results were consistent with our previous behavioral study (Katsuyama et al., 2018): the perceived hardness significantly increased as the hardness of the pad in the mirror reflection increased in the SYNC condition and remained constant in the ASYNC condition (Supplementary Figure 1). This result indicated that the visual modulation of hardness perception was induced even while the participant was lying in the MR scanner. To acquire enough number of trials with visual modulation for multivariate analysis, we used only the SYNC condition in the main experiment (see the Discussion section for details).

#### Main Experiment

**FIGURE 2:**
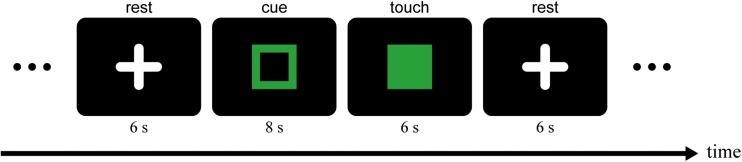
Schematic diagram of the hardness estimation task. The time sequence of one trial is depicted. An experimenter beside the scanner gave a pad to each of the participants’ hands during the cue period. They were required to touch the pad with both hands simultaneously while watching the mirror reflection of the left hand during the touch period. In the following rest period, the experimenter removed the pads, and participants answered the hardness of the pad perceived by the right (hidden) hand orally. During the rest period, they were required to fixate the fixation target (a white cross).

During scanning runs, participants were required to perform a hardness estimation task. Figure 2 illustrates the schematic diagram of the task. The task was performed in a block design, and the scanner and a computer for the behavioral task were synchronized by a trigger signal from the scanner. Each run began with an initial rest (14 s). In the initial 10 s of the rest, participants were instructed to read Japanese characters presented on the monitor aloud for the sensitivity adjustment of the voice-capture system in the scanner noise. After the initial rest, an open square was presented on the monitor for 8 s (cue period). During the cue period, an experimenter beside the scanner presented a pad to each of the participants’ hands. To the left hand, one of three pads (S, M, or H) was presented in a given trial, while only M was presented to the right hand in all trials. Participants were instructed to place the middle three digits on the top surface of the pad and hold them. When the open square was replaced with a solid square on the monitor, the participants touched the pad with both hands while observing the reflection of the left hand for 6 s (touch period). The task was to estimate the hardness of the pad perceived with the right (hidden) hand during the period. Participants were told to investigate how hard (soft) the pad felt, not to estimate the physical hardness of the pad. At the end of the touch period, the experimenter removed the pads from the participants’ hands, and they reported the estimated hardness based on magnitude estimation (Gescheider, 1997), such that the impression of the hardness of the pad matched the size of the positive number. Any positive numbers, including decimals and fractions, could be used for the estimation. Assuming participants would make an absolute ratio judgment of sensory magnitude (Gescheider, 1997), we did not use a standard stimulus (modulus) in the present study. Furthermore, no limitation was employed for the maxima and minima of the numbers. The responses were recorded and served as the off-line analysis. After another rest period (6 s), the subsequent cue period followed. In all rest periods, the participants were asked to look at a white cross presented on the monitor in the mirror on the head coil. They were also instructed to put their hands on the platform in a rest position during the period. Before data collection began, participants received enough training to be able to make a stable hardness estimation while lying in the scanner.

#### Triplet-Based Presentation

**FIGURE 3:**
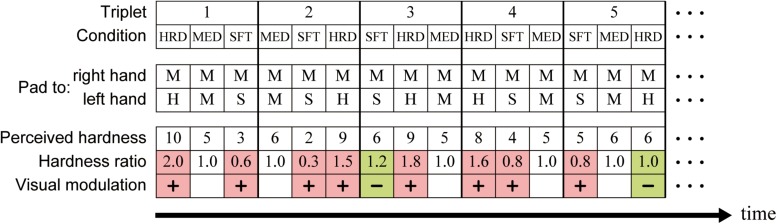
Schematic description of the triplet-based presentation of the stimulus. The tactile stimulation was presented such that any one of conditions SFT, MED, and HRD was assigned to three successive trials in a pseudorandomized order. The hardness ratio was calculated by dividing the perceived hardness in a trial of conditions SFT and HRD by that in condition MED (control) within a given triplet. For example, hardness ratio of the first (condition HRD) and third trial (condition SFT) in the triplet 1 is 2.0 (10/5) and 0.6 (3/5), respectively. Trials with a hardness ratio of <1 in condition SFT and >1 in condition HRD were defined as visually-modulated (colored in pink). In contrast, trials with a hardness ratio of ≥1 in condition SFT and ≤1 in condition HRD were defined as not-visually-modulated (colored in green). HRD, condition HRD; MED, condition MED; SFT, condition SFT; H, hard pad; M, medium pad; S, soft pad.

In the following, the condition (trial) in which pads S, M, and H were presented to the participants’ left hands is referred to as condition SFT, MED, and HRD, respectively. The order of the three conditions was arranged in a triplet-based manner: any one of conditions SFT, MED, and HRD was assigned to three successive trials in a pseudorandomized order (Figure 3). One run consisted of eight triplets, which resulted in 24 trials in a run (eight trials/condition). Six triplets^1^ were arranged into overall runs and assigned to participants in a pseudorandomized order. Each participant completed eight runs (64 trials/condition/participant).

### Imaging Data Acquisition

Images were acquired using a 3T scanner (Magnetom Trio, Siemens, Erlangen, Germany) at Tamagawa University Brain Science Institute (Machida, Tokyo, Japan). We obtained functional blood oxygen level dependent (BOLD) images during the hardness estimation task [T2^∗^-weighted gradient-echo echo-planar images: 34 slices, whole brain, repetition time (TR) = 2000 ms, echo time (TE) = 25 ms, in-plane resolution = 3 × 3 mm, slice thickness = 3 mm, 25% inter slice gap, resultant voxel size = 3 × 3 × 3.75 mm, descending order, flip angle = 75°, field of view (FOV) = 192 × 192 mm]. During one run of the functional scan (502 s), 251 volumes were collected. We also collected structural MRI data for individuals (T1-weighted Magnetization Prepared Rapid Acquisition GRE: 192 sagittal slices, whole brain, TR = 2000 ms, TE = 1.98 ms, in-plane resolution = 1 × 1 mm, slice thickness = 1 mm, flip angle = 10°, FOV = 256 × 256 mm).

### Data Analysis

#### Behavioral Data

Because the visual and haptic information was incongruent in conditions SFT and HRD, but congruent in condition MED, we expected visual information to modulate hardness perception in conditions SFT and HRD, but not in condition MED. Therefore, condition MED was used as the control, and visual modulation was quantified by dividing the perceived hardness in a trial of conditions SFT and HRD by that in condition MED within a given triplet (the hardness ratio). A hardness ratio of <1 in condition SFT and >1 in condition HRD indicated that participants considered the pad to be softer and harder than that of the control (condition MED), respectively. We defined these trials as visually-modulated, and trials in condition SFT with a ratio of ≥1 and those in condition HRD with a ratio of ≤1 as not-visually-modulated (Figure 3). Finally, we also calculated the incidence of visually-modulated trials in conditions SFT and HRD in every run (modulation ratio).

#### MRI Data

##### Multivariate analysis

Preprocessing was performed using the SPM8 software package (Wellcome Trust Centre for Neuroimaging)^2^. The volumes of the initial rest period were discarded. Motion correction was performed by realigning all volumes to the mean of the functional images for each participant followed by slice timing correction. Parameters for co-registration of anatomical images to the mean functional image were calculated and saved for each run. After preprocessing, we ran individual general linear model (GLM) analyses to estimate the cortical activity to the following six conditions per run: (1) SFTvm: visually-modulated trials in condition SFT, (2) SFTnm: not-visually-modulated trials in condition SFT, (3) HRDvm: visually-modulated trials in condition HRD, (4) HRDnm: not-visually-modulated trials in condition HRD, (5) MED: trials in condition MED, and (6) NULL: missed trials. The head motions were additionally modeled as “of no interest” conditions along with condition NULL.

An MVPA was carried out using The Decoding Toolbox (TDT; Hebart et al., 2015) and custom-written programs written in MATLAB (The MathWorks, Natick, MA, United States). A classification accuracy map was calculated for the pair-wise comparisons of (SFTvm vs. MED), (HRDvm vs. MED), and (SFTvm vs. HRDvm) using the GLM parameter estimates for each of the three conditions SFTvm, HRDvm, and MED. A whole-brain searchlight analysis (Kriegeskorte et al., 2006) was performed using 9 mm-radius spheres centered around a given voxel for all voxels. A support vector machine (LIVSVM; Chang and Lin, 2011) was trained on seven out of the eight runs and tested on the untrained run, yielding a classification accuracy map. This process was performed iteratively until all runs had been tested (a leave-one-run-out cross-validation scheme), and a mean accuracy map was calculated by averaging the eight accuracy maps for each of the three comparisons.

Group analysis was performed using SPM8. The mean accuracy maps for the three comparisons were individually normalized to the standard Montreal Neurological Institute (MNI) brain template using the parameters obtained by the co-registration, and smoothed with an 8 mm full-width-half-maximum (FWHM) isotropic Gaussian kernel. The normalized and smoothed maps were entered into a random-effect analysis to investigate the voxels with significantly higher accuracy than chance using a one-way within-subject ANOVA implemented in the second-level routine of SPM8. The resultant statistical map of *T*-value for the comparisons of (SFTvm vs. MED), (HRDvm vs. MED), and (SFTvm vs. HRDvm) was pseudocolored and overlaid on the structural image. To isolate brain regions encoding the perceived hardness with visual modulation, we performed conjunction analysis of the three *T*-value maps against the conjunction null hypothesis (Nichols et al., 2005). This analysis seeks the significant voxels present in all three maps. For the group analysis, a statistical threshold of *p* < 0.05 corrected for familywise error rate was applied. No extent threshold was used. The peak coordinates of the resultant clusters were represented in the MNI coordinate system. The locations of the clusters were identified using an anatomical toolbox for SPM8 (SPM Anatomy Toolbox v. 2.2b, Eickhoff et al., 2005) and a human brain atlas (Talairach and Tournoux, 1988).

##### Univariate analysis

All analyses were performed using SPM8. The preprocessing was conducted as follows: Motion correction by realigning all volumes to the mean functional image, slice timing correction, co-registering of the anatomical image to the mean functional image, normalization of the all functional images to the MNI template, and spatial smoothing with an 8 mm FWHM isotropic Gaussian kernel.

After preprocessing, we ran individual GLM analyses using the same conditions as in the multivariate analysis (SFTvm, SFTnm, HRDvm, HRDnm, MED, NULL, and the realigned parameters) to estimate the cortical activity per condition. For the group analysis, contrast images of conditions SFTvm, HRDvm, and MED were entered into second-level random-effect analysis by a one-sample *t*-test on a voxel-to-voxel basis, and the cortical regions activated by contrasts (SFTvm > MED), (HRDvm > MED), (SFTvm > HRDvm), and (HRDvm > SFTvm) were investigated. We also performed a correlation analysis. We then ran further individual GLM analyses using the following parameters as the parametric modulation: (1) The physical hardness of the pad. This analysis was conducted to identify the cortical regions that exhibited activity changes with varying hardness of the pad regardless of visual modulation. Two conditions, PAD and NULL, were employed. The condition PAD indicates the sum of conditions SFTvm, SFTnm, HRDvm, HRDnm, and MED. The physical hardness of the three pads described earlier was entered as the parametric modulation of all trials of condition PAD, and the contrast images were used for the population analysis; (2) the raw values of the perceived hardness; and (3) the hardness ratio. These analyses were performed to identify the cortical regions that exhibited activity that correlated with the perceived hardness with visual modulation. Four conditions, VM, NM, MED, and NULL, were employed. Conditions VM and NM indicated the sum of conditions SFTvm and HRDvm, and SFTnm and HRDnm, respectively. The behavioral parameters were entered as the parametric modulation. A population analysis was conducted using the contrast images of condition VM. A statistical threshold of *p* < 1 × 10^–5^, uncorrected for multiple comparisons, was applied for the group analysis. An extent threshold >50 voxels was used.

### Hand Ownership and Agency Test

After MRI, a questionnaire regarding the ownership and agency of the mirror image of the hand was administered. The items were invented with reference to previous studies (Botvinick and Cohen, 1998; Longo et al., 2009; Kalckert and Ehrsson, 2014; Kokkinara and Slater, 2014; Jenkinson and Preston, 2015; Ma and Hommel, 2015).

The items were as follows:

1. It felt like I was looking directly at my hand rather than at a mirror image.
2. It felt like the hand I was looking at was my hand.
3. Did it seem like the hand you saw was a right hand or a left hand?
4. The movements of the hand in the reflection were caused by my movements.
5. The hand in the reflection moved just like I intended to.
6. Was the hand you intended to move a right hand or a left hand?
7. I felt as if I no longer had a right hand, as if my right hand had disappeared.
8. It seemed as if I might have more than one right hand.
9. I felt as if the movements of my right hand were controlled by someone else.
10. I felt as if the hand in the reflection was controlling my movements.
11. I felt like my right hand was located at a different position to that in the reflection.

The questionnaire items were translated into Japanese. The participants were required to rate their agreement with each item on a seven-point scale ranging from −3 (strongly disagree) through 0 (neither agree nor disagree) to +3 (strongly agree). For items 3 and 6, a different scale ranging from −100 (strongly left hand) through 0 (equally left and right hands) to +100 (strongly right hand) was used. Any intermediate value could be used. Agreement or disagreement was tested by comparing the mean score to 0 using a one sample *t*-test at a significance level of *p* < 0.05. Furthermore, the participants answered the following questions in a post-experimental interview:

1. Did you know how many different pads were presented to each of your hands in all runs?
2. Which hand was presented with a wider range of pads of varying hardness; your right hand or your left hand?
3. Did you find that the physical hardness of the pad presented to your right hand was constant throughout all runs?

## Results

### Results of the Hardness Estimation Test During Scanning

**FIGURE 4:**
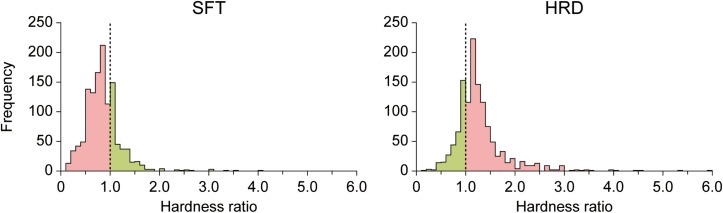
Distribution of the hardness ratio across all participants and runs. The visually-modulated trials in conditions SFT and HRD were defined as conditions SFTvm and HRDvm, respectively (colored in pink). In contrast, the not-visually-modulated trials in conditions SFT and HRD were defined as conditions SFTnm and HRDnm, respectively (colored in green).

In the present study, condition MED was used as a control to test for visual modulation in conditions SFT and HRD. Thus, the ratios of perceived hardness SFT/MED and HRD/MED were calculated for each triplet (hardness ratio) and used to determine whether the perceived hardness was modulated by visual information in a given trial. Figure 4 illustrates the distribution of the hardness ratio in conditions SFT and HRD across all participants and runs. Trials of condition SFT with hardness ratios of <1 indicated that the pad was perceived to be softer than the control. Likewise, trials of condition HRD with ratios >1 indicated that the pad was perceived to be harder than the control. We then defined these trials as visually-modulated (SFTvm and HRDvm). The ratio of the visually-modulated trials in conditions SFT and HRD (modulation ratio) averaged over all eight runs for each participant is provided in Table 1. The mean modulation ratio was 73.4% ± 17.7% and 74.0% ± 18.2% for conditions SFT and HRD, respectively. There was no significant difference between these ratios [Welch’s *t-*test, *t*_(42)_ = 0.11, *p* = 0.91]. Furthermore, we calculated the modulation ratio of the SFT and HRD conditions for each of the six triplets overall runs and the participants with two-way repeated measures ANOVA, for computing the main effects of the condition (SFT vs. HRD) and the triplet (6 triplets), and found no significant difference between the ratios [*F*_(1, 21)_ = 0.08, *p* = 0.78 for the main effect of the condition; *F*_(5, 105)_ = 1.73, *p* = 0.13 for the main effect of the triplet; and *F*_(5, 105)_ = 0.83, *p* = 0.53 for the interaction between the main effects]. The modulation ratios for each participant, condition, and run are provided in Supplementary Table 1.

**TABLE 1:** Modulation ratio.

**Participant**	**SFT**	**HRD**
1	96.9	60.9
2	57.8	57.8
3	87.5	92.2
4	93.8	92.2
5	89.1	65.6
6	62.5	45.3
7	89.3	92.9
8	43.8	54.7
9	67.2	90.6
10	50.0	57.8
11	100.0	95.3
12	53.6	85.7
13	51.6	49.6
14	87.5	96.9
15	83.9	85.7
16	89.1	100.0
17	90.6	59.4
18	76.6	53.1
19	48.4	59.4
20	75.0	57.8
21	60.9	89.1
22	59.4	85.9
Mean ± SD	73.4 ± 17.7	74.0 ± 18.2

**The incidence of the visually-modulated trials in the conditions SFT and HRD averaged over the 8 runs for each participant is shown (%). SD, standard deviation.**

The post-experimental interview revealed that most participants did not find that the physical hardness of the pad presented to the hidden hand was constant during scan, until they were informed of the “gimmick” of the task after the MRI experiment. However, two participants were vaguely aware of the possibility. The number of pads of different degrees of hardness presented to the left and right hand was 6.3 ± 2.4 and 6.0 ± 3.5, respectively. Although there was no significant difference between the number of different pads presented to each hand [Welch’s *t*-test, *t*_(42)_ = 0.35, *p* = 0.73], most participants (15/22) felt that a wider range of pads of varying hardness was presented to the left hand than to the right hand.

### Results of the fMRI Multivariate Analysis

**FIGURE 5:**
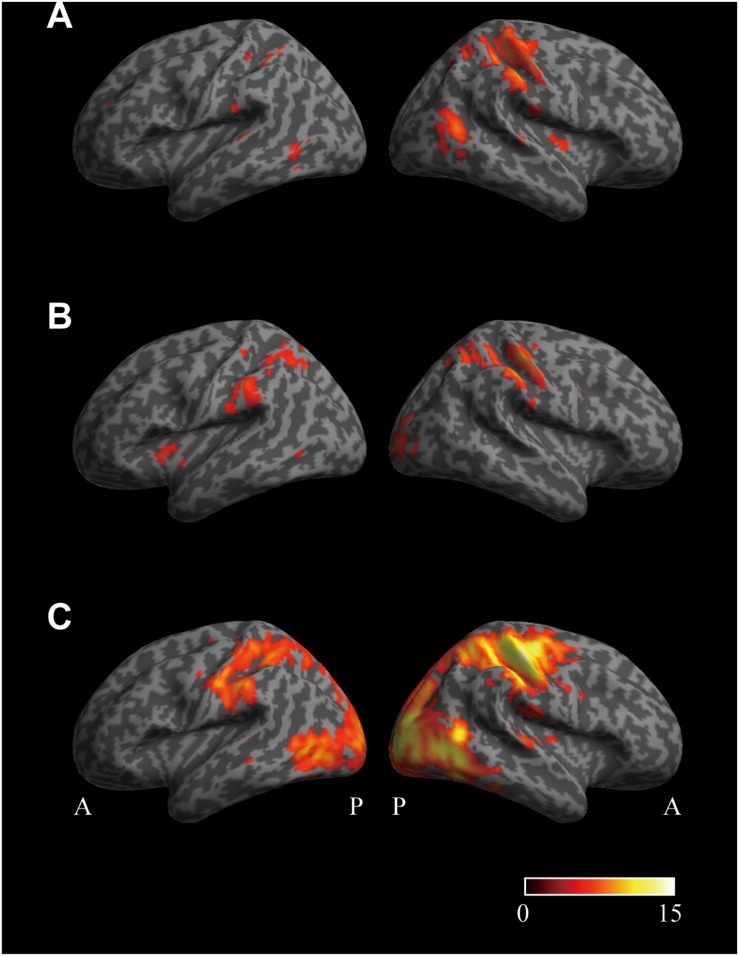
Cortical regions related to the perceived hardness obtained by multivariate analysis. **(A)** The surface view of the cortical regions encoding the perception that the pad is softer than the control condition. The cortical regions whose activity patterns were different between the conditions SFTvm and MED were investigated by multivariate analysis. **(B)** Brain regions encoding the perception that the pad is harder than the control condition. The areas whose activity patterns were different between the conditions HRDvm and MED were investigated. **(C)** Cortical regions encoding the perceived hardness. Brain areas whose activity patterns were different between the conditions SFTvm and HRDvm were investigated. The lateral view of the left and right hemisphere is shown in the left and right column, respectively, in all panels. The significance level was set at *p* < 0.05 corrected for familywise error rate. No extent threshold was applied. The color bar indicates the *T*-value. A, anterior; P, posterior.

Cortical regions encoding the perception that the pad is softer than the control were investigated by multivariate analysis using the pair-wise comparison of (SFTvm vs. MED) (Figure 5A). A large cluster was observed in the sensory motor cortex (SMC) extending from the precentral gyrus to the intraparietal sulcus (IPS), parietal operculum (PO), insula, and occipito-temporal cortex (OTC) of the right hemisphere. In the left hemisphere, a cluster was observed in the anterior IPS (aIPS), PO, and OTC. There were no clusters found in the left SMC.

Similarly, the brain regions encoding the perception that the pad is harder than the control were investigated by the comparison of (HRDvm vs. MED) (Figure 5B). A large cluster was observed in the SMC, PO and visual areas in the occipital cortex of the right hemisphere. In the left hemisphere, a cluster was found in the anterior insula, aIPS, PO, and OTC. There were no clusters obtained in the left SMC either.

Finally, differences in perceived hardness between SFT and HRD conditions were investigated by the comparison of (SFTvm vs. HRDvm) (Figure 5C). In this analysis, a large cluster covering the motor, sensory, and visual cortices was observed in the right hemisphere. Similarly, a large cluster was obtained in the left hemisphere as well, however, only a few clusters were found in the left motor cortex.

**FIGURE 6:**
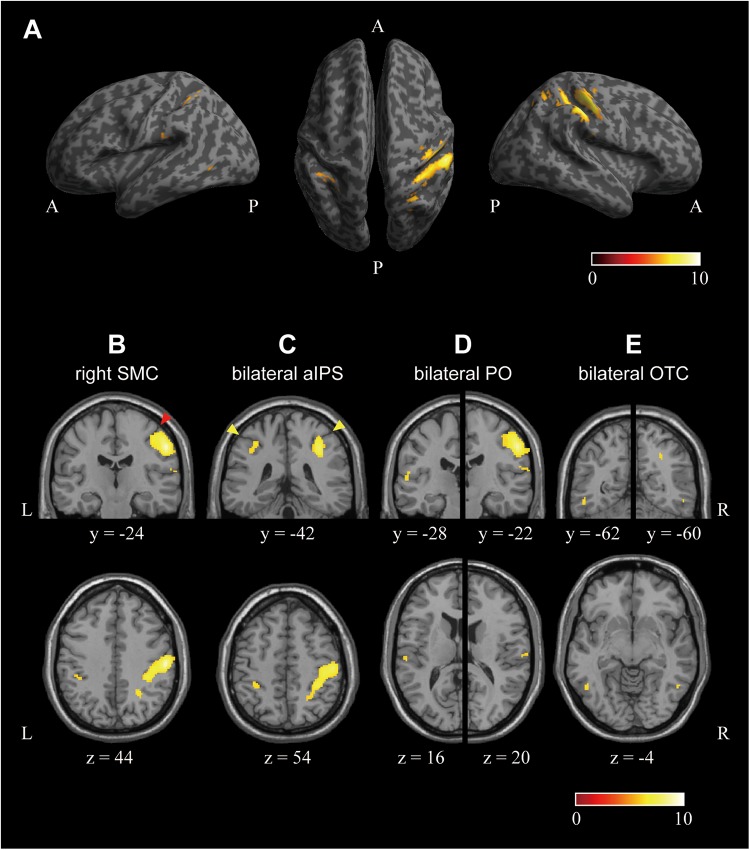
Cortical regions encoding the perceived hardness with visual modulation. Regions encoding the perceived hardness with visual modulation were investigated by conjunction analysis of the three comparisons of (SFTvm vs. MED), (HRDvm vs. MED), and (SFTvm vs. HRDvm). **(A)** The surface view. Left: lateral view of the left hemisphere, Right: lateral surface of the right hemisphere, Middle: top view of both hemispheres. **(B)** A cluster observed in the right sensory motor cortex (SMC). The coronal section at the local maxima identified in S1 is shown. **(C)** Clusters observed in the anterior intraparietal sulcus (aIPS) in both hemispheres. The cluster in the right aIPS was part of the large cluster in the SMC. **(D)** Clusters obtained in the parietal operculum (PO) of both hemispheres. **(E)** Clusters in the bilateral occipito-temporal cortex (OTC). The cluster was located in the posterior inferior temporal sulcus. The red and yellow arrow heads in **(B,C)** indicate the central and intraparietal sulcus, respectively. The peak and extent thresholds were the same as in Figure 5. The color bars indicate the *T*-value. A, anterior; P, posterior.

Next, we investigated the cortical regions encoding the perceived hardness with visual modulation by conjunction analysis of the three comparisons of (SFTvm vs. MED), (HRDvm vs. MED), and (SFTvm vs. HRDvm). Figure 6 shows the result. A large cluster extending from the posterior portion of the precentral gyrus to the aIPS through the postcentral gyrus and sulcus was observed in SMC of the right hemisphere (Figures 6B,C). The local maxima of the cluster (x, y, z = 54, -24, 44) was located in the postcentral gyrus (Figure 6B). In the left hemisphere, no cluster was observed in the SMC. However, a cluster was identified in the aIPS (Figure 6C). Another cluster was obtained in the posterior PO (Figure 6D) and OTC (Figure 6E) of both hemispheres. The cluster in the OTC was located in the posterior portion of the inferior temporal sulcus. It is noteworthy that the cluster in the aIPS, PO, and OTC was symmetrical in both hemispheres. The location, size, Brodmann’s area (BA), MNI coordinates, *T*-value, and mean accuracy over all participants of the clusters are indicated in Table 2.

**TABLE 2:** Cortical regions encoding the perceived hardness with visual modulation.

**Position of peaks**	**L/R**	**BA**	**Cluster size**	***T*-value**	**MNI**			**Mean accuracy**		
					**coordinates**			** **				
					**x**	**y**	**z**	**SFTvm vs. MED**	**HRDvm vs. MED**	**SFTvm vs. HRDvm**
SMC	R	1	1462	9.13	54	–24	44	61.7 ± 8.1	61.1 ± 6.8	66.2 ± 8.5
		7		7.82	34	–42	48			
		4		5.97	38	–22	56			
aIPS	L	7	75	6.34	–36	–42	54	61.0 ± 10.0	60.3 ± 8.5	63.1 ± 8.3
PO	L	40	18	6.24	–58	–28	16	58.9 ± 7.6	57.3 ± 7.7	57.2 ± 7.9
PO	R	40	13	6.01	62	–22	20	60.0 ± 8.6	59.3 ± 8.1	59.6 ± 9.6
OTC	L	37	17	6.28	–50	–62	–4	59.6 ± 9.2	58.5 ± 8.9	60.9 ± 8.7
OTC	R	37	6	6.13	50	–60	–4	61.0 ± 10.8	59.5 ± 6.6	67.6 ± 12.2

**For the large cluster in the SMC of the right hemisphere, the location of the sub-peaks is shown, as well as the local maxima. The mean accuracy was calculated over all participants for each of the clusters. L, left hemisphere; R, right hemisphere; BA, Brodmann’s area; MNI, Montreal Neurological Institute; aIPS, anterior intraparietal sulcus; OTC, occipito-temporal cortex; PO, parietal operculum; SMC, sensory motor cortex.**

### Results of the fMRI Univariate Analysis

Univariate analysis was performed using the same data set as was used in the multivariate analysis. First, we analyzed the contrast images of (SFTvm > MED) and (HRDvm > MED) to identify the cortical regions that were more strongly activated in the visually-modulated conditions than in the control. However, we did not observe any activation at the threshold in the population analysis. No activation was observed in the contrast images of (SFTvm > HRDvm) and (HRDvm > SFTvm) either. We then applied a correlation analysis to investigate the cortical regions that exhibited altered activity with varying physical hardness of the pad irrespective of visual modulation. Some participants exhibited significant activation with a BOLD signal that was negatively correlated with the hardness of the pad in the right SMC. However, no significant cluster was obtained in the population analysis of all participants. Finally, we attempted to identify the cortical regions wherein the activity correlated with the perceived hardness modulated by visual information, by entering the raw values of the perceived hardness or hardness ratio as the parametric modulation, but found no significant activation. Thus, we did not obtain any significant activation related to the physical or perceived hardness of the pad in the univariate analysis in the present study.

### Results of the Hand Ownership and Agency Test

**FIGURE 7:**
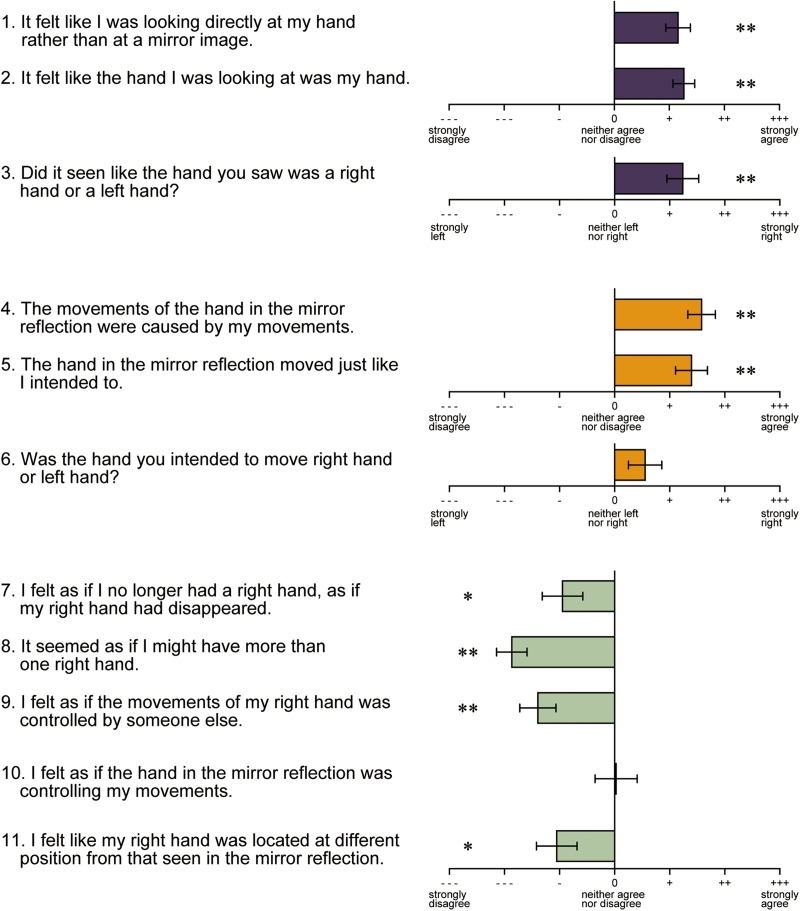
Results of the hand ownership and sense of agency test. Items 1–3 and 4–6 in the questionnaire were related to illusory hand ownership and sense of agency, respectively. The other items are the controls. Error bar: standard error (S.E.). ^∗∗^*p* < 0.01, ^∗^*p* < 0.05.

After the fMRI experiment, a questionnaire regarding illusory hand ownership and sense of agency toward the hand reflection was administered (Figure 7). Items 1–3 were related to illusory ownership. The results revealed that the vast majority of the participants felt that the reflection of the left hand was their own right hand. Items 4–6 were related to the sense of agency. Participants reported that the motion of the hand in the reflection was the motion intended for their own hand. However, the score of item 6, which asked whether the motion of the reflected hand was the one they intended to make with their right or left hand, was biased toward the right hand, but not significantly. The other items (7–11) were the control questions to items 1–6. However, the participants awarded significantly negative scores to all items with the exception of item 10. These results indicate that, although the participants felt that the hand in the reflection was their own right hand and the motion was caused by their own will, it was not necessarily the motion they intended to make with the right hand.

## Discussion

### Hardness Perception Modulated by Visual Information

The aim of the present study was to identify the cortical regions involved in hardness perception modulated by visual information. The participants performed the hardness estimation task using MVF while lying in an MR scanner. They were required to estimate the hardness of a pad presented to the right hand that was placed behind the mirror while observing the reflection of the left hand pushing a pad simultaneously. To the left hand, one of three pads (soft, medium, and hard) was presented in a given trial (condition SFT, MED, and HRD, respectively), whereas only the medium pad was presented to the right hand across all trials. The behavioral result indicated that the participants felt that the pad presented to the hidden hand was softer or harder than the control condition (MED) in >73 and 74% of the trials in conditions SFT and HRD, respectively. This result is consistent with that of our previous study demonstrating that the modulation of hardness perception was induced by observation of the reflection of a hand pushing a pad of different hardness. In the study, we excluded the possibility that the modulation was induced by sensory assimilation and bimanual coordination by demonstrating that the modulation diminished when participants touched the pad with both hands at different times or with their eyes closed. Furthermore, no significant difference was observed in the finger displacement of the hidden hand during touch (Katsuyama et al., 2018). In this study, we excluded control conditions to validate the effect of sensory assimilation and bimanual coordination on the behavioral task, to acquire a sufficient number of trials with visual modulation for multivariate analysis, which requires multiple runs (in the present study, participants underwent 192 trials, over eight runs in the supine position). The introduction of control conditions simply doubles the number of total trials. This might cause deterioration of the quality of data due to fatigue. Furthermore, it was impossible to measure the finger movements in the MRI scanner using the electromagnetic tracker used in the previous study. However, we considered the effects of sensory assimilation and bimanual coordination negligible, based on the results of the pilot study (Supplementary Material). The study was conducted to examine whether visual modulation can be induced in a supine position in an MR scanner. Based on our previous study, two touch timing conditions (ASYNC and SYNC) were employed, along with the pad-hardness condition. Since earlier studies have shown that sensory assimilation can be induced when two stimuli are presented to participants sequentially as well as concurrently (Gescheider, 1997; Roberts, 2013), if the modulation of hardness perception was induced by the assimilation of tactile input from both hands, modulation should have been observed in the ASYNC condition also, in which participants touched the pads of varying hardness with both hands in an alternating fashion. However, similar to our previous study, modulation was induced in the SYNC condition, but not in the ASYNC condition, in the pilot study. Although we lack explicit evidence, we believe that it is unlikely that participants used different strategies and/or moved the fingers in a different manner to reproduce the same behavioral result in the upright and supine positions. Therefore, we concluded that the modulation of perceived hardness in the present study was induced by observation of the reflection, even in the supine position.

### Result of the Univariate Analysis of the fMRI Data

In the present study, we analyzed the same dataset with a uni- and multivariate approach. First, we investigated whether there were any cortical regions where the activity was higher in the visually-modulated conditions than in the control condition by subtraction analysis. However, we did not identify any cortical regions. Next, we tried to investigate cortical regions that exhibit activity that correlates with the physical hardness of the pad by correlation analysis. However, no such region was found, either. In the individual analysis, we observed significant activity in the right SMC that was negatively correlated with the hardness of the pad in some participants. Since the finger displacement to push the pad increased as the physical hardness of the pad decreased, it is plausible that the activity reflected the difference in the motor commands for the finger movements and the sensory inputs to the left hand when touching pads of varying hardness during the scan. However, the activity in the right SMC did not exceed the threshold in the group analysis. To the contrary, as detailed below, the informative clusters were obtained in several cortical regions including the right SMC by multivariate analysis. The reason why no activity was found by univariate analysis is not straightforward, because little is known about how hardness (softness) perception is encoded by neurons in the somatosensory cortex. The firing rate of SA1 fibers, which convey the information of the compressional force to the fingers, increases as the indentation of the skin increases (Mountcastle et al., 1966; Harrington and Merzenich, 1970; Muniak et al., 2007). This response property is kept in some S1 neurons (Bensmaia et al., 2008), and may be detectable by univariate analysis. However, cumulative evidence reveals that information processing in S1 is more complicated than thought. Convergence of the information conveyed by different mechanoreceptor afferents including SA1, rapidly adapting (RA)^3^, and Pacinian (PC)^4^ fibers takes place as early as in area 3b, the primary target of the mechanoreceptor afferents from periphery, and proceeds along to areas 1 and 2 (for review, see Delhaye et al., 2018). The ensemble of the various neuronal responses may lead to pattern coding, which is more detectable by multivariate than univariate analysis.

The inconsistency in the activation patterns identified by uni- and multivariate approaches was also reported for stickiness perception (Kim et al., 2017; Yeon et al., 2017). As described earlier, hardness perception is determined by the compressional force to the skin, and the information is conveyed by SA1 fibers from periphery (Srinivasan and LaMotte, 1995; Friedman et al., 2008). Likewise, the sense of stickiness is evoked when the skin is stretched by an adhesive object, and the information about the skin deformation is conveyed by several mechanoreceptor afferents including SA1, slowly adapting type 2 (SA2)^5^, and RA fibers (Yeon et al., 2017). How sensory perception, such as hardness and stickiness, is encoded by cortical neurons and how the neuronal representation is converted to a BOLD signal utilized by fMRI are important problems in the study of the sensory system.

### Result of the Multivariate Analysis of the fMRI Data: The Right Hemisphere

In the present study, we investigated cortical regions involved in the perceived hardness modulated by visual information by using a whole-brain searchlight MVPA (Kriegeskorte et al., 2006). For this purpose, the accuracy map of pair-wise comparisons of (SFTvm vs. MED), (HRDvm vs. MED), and (SFTvm vs. HRDvm) were calculated individually, and the maps were used for conjunction analysis in the group analysis. This analysis seeks the significant voxels present in all three maps. As described earlier, the condition MED was designated as the control to conditions SFTvm and HRDvm for the visual modulation. Therefore, the *T*-value map of the pair-wise comparison of (SFTvm vs. MED) – showing brain regions whose activity patterns are significantly different between conditions SFTvm and MED – indicates cortical regions encoding the perception that the pad is softer than the control in the group analysis. Likewise, the map of (HRDvm vs. MED) illustrates the brain regions encoding the perception that the pad is harder than the control. On the other hand, the *T*-value map of (SFTvm vs. HRDvm) shows the brain regions sensitive to the difference in the perceived hardness. In the present study, we defined such cortical regions as areas encoding the perceived hardness. However, the map also includes those cortical regions used in perceiving hardness without visual input. Therefore, to isolate brain regions involved in integrating visual inputs to perceive hardness from those that do not, we performed conjunction analysis of the three *T-*value maps of (SFTvm vs. MED), (HRDvm vs. MED), and (SFTvm vs. HRDvm).

One may argue that the individual accuracy map should be calculated for the pair-wise comparisons of (SFTvm vs. SFTnm) and (HRDvm vs. HRDnm), because the perceived hardness was different between the conditions, whereas the physical stimuli were the same. However, this analysis proved difficult. The modulation ratio was more than 73% in both conditions SFT and HRD, and the modulated trials of both conditions (SFTvm and HRDvm) were contained in all runs and participants. On the other hand, the trials of conditions SFTnm and HRDnm were not in all runs: the mean number of runs with trials of conditions SFTnm and HRDnm was 6.2 and 6.0 out of 8 runs (range: 0–8 for both conditions) per participant, respectively. If the SVM is trained on a condition pair with an unbalanced number of runs, the labeling by the SVM in the untrained run will be biased toward the condition with the most runs (SFTvm and HRDvm in this case), resulting in deterioration of classification accuracy. Therefore, we restricted our analysis to the comparisons of (SFTvm vs. MED), (HRDvm vs. MED), and (SFTvm vs. HRDvm) in the present study.

The searchlight MVPA revealed a large cluster in the SMC of the right hemisphere. The peak voxel of the region was in the postcentral gyrus (S1), and it extended into the precentral gyrus (M1) rostrally and to IPS caudally through the postcentral sulcus, and to the PO ventrally. In the hardness estimation task, the participants touched pads of varying physical hardness with the left hand. Therefore, it is clear that the large cluster in the right hemisphere reflected cortical activity related to the motor command of the finger movements and sensory response to the tactile and proprioceptive inputs of the left hand. The cluster extended from the postcentral gyrus to the IPS via the postcentral sulcus, covering Brodmann’s area 1 and 2. Neurons in Brodmann’s area 1 and 2 in monkeys represent several fingers, and the receptive fields were overlapped (Iwamura et al., 1980, 1985a). This observation was also confirmed in the human somatosensory cortex (Kurth et al., 2000). These characteristics of the receptive fields of neurons in Brodmann’s area 1 and 2 form a functional surface suitable for various aspects of active touch, such as touching, pinching, and grasping (Iwamura et al., 1985b). As described earlier, when one pushes objects of varying hardness, finger displacement decreases as the physical hardness of the object increases, and the peak compressional force to fingers and perceived hardness would also co-vary with the displacement. Thus, the brain activity pattern obtained in the right hemisphere was depictive of the cortical activity related to the motor commands and somatosensory responses of the left hand in the hardness estimation task.

Contrary to the findings in the right hemisphere, no clusters were found on M1 and S1 of the left hemisphere. Considering that the finger displacement of the right hand was constant across all trials, this result was not unexpected. One possible disadvantage of the task design of the present study is that it may have underestimated the role of S1 in hardness perception. Although no cluster was obtained on the left S1, we cannot exclude the possibility that S1 may play a role in the perception of hardness with visual modulation, because recent studies have suggested that S1 is involved in illusory hand ownership and tactile perception in humans and animals (Chen et al., 2003; Blankenburg et al., 2006; Schaefer et al., 2006; Hötting et al., 2009; Shokur et al., 2013). In the present study, participants actually touched a pad with their right hand. The continuous tactile input from the periphery might mask the illusion-related signal in S1 and make it difficult to detect by either univariate or multivariate analysis. The absence of activity in S1 in illusory ownership and tactile perception has been observed in previous fMRI studies, in which participants received tactile stimulation in both test and control conditions (for example, Ehrsson et al., 2004; Matthys et al., 2009; Chambon et al., 2013; Kitada et al., 2014a; Limanowski et al., 2014). However, when a tactile illusion is induced on a part of the body, by tactile stimulation delivered to another part of the body and at different times, the illusion-related signal is detected on the somatotopically corresponding site at S1 by fMRI (Blankenburg et al., 2006).

Although sensitivity to S1 might be low, considering the difference in activation patterns between the right and left SMC, we concluded that the cortical regions encoding the perception of hardness with visual modulation can be successfully isolated from those involved in the motor commands, using the hardness-estimation task and multivariate analysis as described in the Introduction.

### Result of the Multivariate Analysis of the fMRI Data: The Bilateral aIPS

A cluster was found in the aIPS of the left hemisphere. It is well known that the IPS plays a crucial role in multimodal integration. Recent neuroimaging studies have demonstrated that visual, auditory, somatosensory, and vestibular inputs converge in the IPS of both humans and macaque monkeys (Guipponi et al., 2013; Huang and Sereno, 2018). In monkeys, it has been revealed that neurons in the bottom of the intraparietal sulcus (ventral intraparietal area, area VIP) respond to both visual and tactile stimuli (Colby and Duhamel, 1991; Duhamel et al., 1998), and that the activity of some bimodal neurons in VIP is higher when the visual and tactile stimulation was presented at the same time than when each stimulus was presented individually (Avillac et al., 2007). It is noteworthy that the cluster in the left aIPS obtained in the present study was located near the bottom of the sulcus. In humans, many studies have demonstrated that the aIPS is commonly activated by visual and haptic cues in shape and texture recognition tasks (Macaluso and Driver, 2001; Kitada et al., 2006; Stilla and Sathian, 2008; Gentile et al., 2011; Joanne Jao et al., 2014). The activity of the aIPS is enhanced by visuo-tactile stimulation that is more than the sum of the visual and tactile stimulation alone (Gentile et al., 2011). The activity of the aIPS is also observed in object discrimination in a delayed-matching-to-sample task in which the sample and test stimuli were presented visually and haptically, respectively, and vice versa (Grefkes et al., 2002; Kassuba et al., 2013). Pasalar et al. (2010) reported that application of transcranial magnetic stimulation (TMS) to the aIPS disrupts visuo-tactile integration. They found that the detection of tactile stimulation in a finger is enhanced by observation of a visual cue pointing to the same finger. However, the enhancement is eliminated by the application of TMS to the junction of the postcentral sulcus and the aIPS.

The aIPS and posterior parietal cortex (PPC) is also known as a higher order somatosensory cortex. Activation of the aIPS is observed in haptic object (shape) recognition tasks (Pietrini et al., 2004; Hinkley et al., 2009; Podrebarac et al., 2014; Hernández-Pérez et al., 2017) and vibration stimulation to a finger (Schmidt et al., 2014). With regard to the present study, Leib et al. (2016) suggested that stiffness perception may be represented in the PPC. In their study, participants estimated the stiffness of a surface projected on a display by “pushing” the virtual surface with the hand via a haptic device. They applied theta-burst TMS to the PPC of participants and demonstrated that the stiffness perception was disrupted by TMS but the hand movements were not. Moreover, Newman et al. (2005) reported that imagery of the material dimension of objects (roughness, hardness, and temperature) activated the posterior IPS. Finally, it is noteworthy that the cluster was observed in the corresponding site of the aIPS in both hemispheres in the present study. In the hardness estimation task, participants actually touched the pads of varying physical hardness with the left hand, and touched the pad of constant hardness with the right hand. Nevertheless, the perceived hardness of the pad presented to the right hand was modulated by visual information. Thus, the present result that the cluster was located in the corresponding site of the aIPS suggests that this region may be involved in the perception of hardness. Taken together, these results indicate that the aIPS plays a crucial role in not only the integration of visual and haptic information, but also in hardness perception in the present study.

### Result of the Multivariate Analysis of the fMRI Data: The Bilateral PO

A cluster was observed in the PO of both hemispheres. This region is known to be the anatomical site of somatosensory area 2 (S2). Human PO is divided into four cytoarchitectonically distinct sub-regions termed OP1 – 4. On the basis of the somatotopic arrangement in the lateral fissure, Eickhoff et al. (2006) suggested that areas OP1, OP3, and OP4 may be the human homologs to non-human primate areas S2, ventral somatosensory (VS; Cusick et al., 1989), and parietal ventral (PV; Krubitzer et al., 1995), respectively. The cluster obtained in the present study was located in the posterior and lateral portion of PO in both hemispheres. On the basis of the topography of the four sub-regions in PO (Eickhoff et al., 2006), the cluster may be in OP1 (S2).

Several studies have shown that human PO is involved in somatosensory processing. Electrical stimulation to the Sylvian fissure evokes somatosensory sensations (Penfield and Jasper, 1954; Lüders et al., 1985). Clinical studies have reported that a lesion in PO impairs somatic sensation including pain (Caselli, 1991; Bowsher et al., 2004). By using an fMRI technique, Servos et al. (2001) also found that PO is activated by the hardness identification task, although the location was more anterior to the cluster obtained in the present study. Furthermore, activation of PO has been observed by tactile or electrical stimulation to the hand via fMRI (Disbrow et al., 2000; Ruben et al., 2001; Eickhoff et al., 2007; Hinkley et al., 2007; Burton et al., 2008; Mazzola et al., 2012; Tamè et al., 2012; Björnsdotter et al., 2014; Morrison, 2016), EEG (Stančák et al., 2005), and MEG (Disbrow et al., 2001; Lin and Forss, 2002) studies.

It has been suggested that PO (S2) is a higher order area than S1. Physiological studies with monkeys have shown that neurons in S2 have more complex response properties than S1: they have a larger receptive field and respond to both contra- and ipsilateral stimulation; some S2 neurons respond to stimulation to multiple digits; about one-fourth of S2 neurons have their receptive field in more than one of the four body parts (head, trunk, forelimb, and hindlimb); finally, some S2 neurons are silent to passive stimulation but active in active touch task (Sinclair and Burton, 1993; Fitzgerald et al., 2006a, b; Taoka et al., 2016). Bilateral response has been also confirmed in human PO by fMRI and EEG/MEG studies (Disbrow et al., 2001; Eickhoff et al., 2007; Burton et al., 2008; Mazzola et al., 2012; Tamè et al., 2012). Anatomical studies have shown that S2 has connections with the insula, PPC (area 7), motor cortex, and prefrontal cortex in monkeys (Pandya and Kuypers, 1969; Mufson et al., 1981; Friedman et al., 1986; Matelli et al., 1986; Carmichael and Price, 1995). Functional imaging studies have shown that PO has anatomical and functional connectivity with these cortical regions in humans as well (Eickhoff et al., 2010; Mălîia et al., 2018). Binkofski et al. (1999) revealed that PO is involved in object recognition by hand manipulation. In the study, activation of PO was observed without activation of S1 when participants manipulated complex objects. It is noteworthy that a cluster was observed in the left PO while no clusters were found in S1 during the hardness estimation task by active touch in the present study. Activation of PO was also observed in the haptic texture identification task (Stilla and Sathian, 2008). PO is also engaged during a tactile illusion: when the tendon of the wrist extensor muscle is stimulated by vibration, an object touched by the stimulated hand is perceived to move with the hand without any real motion of the hand (hand-object illusion) (Naito and Ehrsson, 2006). Furthermore, PO, but not S1, is activated while watching a movie of someone being touched on the leg. Interestingly, the same region in PO is also activated by tactile stimulation to the same leg of participants (Keysers et al., 2004). The activation observed in the study by Keysers et al. was located close to the cluster obtained in the present study. All these observations indicate that human PO is involved in somatosensory perception, and this region may play a key role in perceiving hardness with visual modulation in the present study.

Considering that PO is sensitive to not only the contralateral but also ipsilateral tactile stimulation, one may argue that the cluster in the left PO may be related to the haptic input from the left hand. This is a valid point, and we do not exclude this possibility. However, we suggest that the region in the left PO also conveys substantial information on the subjective hardness perceived by the right hand for the following reasons. First, although PO is sensitive to tactile stimulation to either side, the response is stronger to contralateral than ipsilateral stimulation in most cases (Burton et al., 1999, 2008; Disbrow et al., 2000, 2001; Ruben et al., 2001; Servos et al., 2001; Stančák et al., 2005; Eickhoff et al., 2007; Mazzola et al., 2012). Second, a large cluster was observed in the right SMC, reflecting that participants actually touched the pads of varying physical hardness with the left hand throughout all trials. To the contrary, no cluster was observed in the left S1, suggesting that very little information is sent from S1 to PO in the left hemisphere. These results predict that more information about the perceived hardness should be processed in the right PO than the left. However, there was no difference in the size and mean accuracy of the cluster in PO between the left and right hemispheres. The most plausible explanation for this discrepancy is that a certain amount of information about the perceived hardness by the right hand is processed in the left PO. It has been shown that human PO has both anatomical and functional connectivity with aIPS (Eickhoff et al., 2010). Taken together with the earlier discussion about the aIPS, it seems reasonable to suppose that the haptic input from the right hand and visual information are integrated in the left aIPS, and the perceived hardness modulated by the visual information is processed in the cortical network between aIPS and PO.

### Result of the Multivariate Analysis of the fMRI Data: The Bilateral OTC

We also observed a cluster in the bilateral OTC. It was located in the inferior temporal sulcus, and the location was symmetrical in both hemispheres. By comparison of the coordinates with those from previous studies, we propose that the region is in the extrastriate body area (EBA). EBA was initially described as one of the extrastriate visual areas that selectively responds to images of body parts except the face (Downing et al., 2001). The mean coordinates of the local maxima of EBA averaged over the previous 11 studies were (x, y, z) = (−47.1 ± 2.6, −72.2 ± 2.7, −3.4 ± 6.0), and (47.1 ± 3.1, −67.9 ± 3.0, −3.1 ± 5.1) in left and right hemisphere, respectively (based on data in Ferri et al., 2013). In comparison, our cluster was located more anteriorly to the classical EBA at the local maxima (left: −50, −62, −4; right: 50, −60, −4). However, the extent of EBA identified by the localizer task (based on the method by Downing et al., 2001) is sometimes larger, covering the anterior occipital to posterior temporal cortices (for example, Spiridon et al., 2006; Orlov et al., 2010; Costantini et al., 2011; Kitada et al., 2014b). Moreover, studies on the topographical organization of EBA showed that the hand is represented anterior to the other body parts, such as the foot and trunk (Astafiev et al., 2004; Orlov et al., 2010). Therefore, the cluster in the OTC may be included in, or overlap with, the anterior portion of EBA that represents the hands somatotopically.

Previous studies have shown that EBA is one of the cortical regions related to illusory hand (body) ownership (Matthys et al., 2009; Limanowski et al., 2014; Limanowski and Blankenburg, 2015), along with the ventral premotor cortex (vPM), right temporo-parietal junction (rTPJ), and PPC (Ehrsson et al., 2004, 2005; Arzy et al., 2006; Tsakiris et al., 2008; Gentile et al., 2011; Ionta et al., 2011; Brozzoli et al., 2012). Using MVF, Matthys et al. (2009) found activation in the OTC when participants observed a mirror reflection of one hand while the other hand was hidden behind the mirror. Because participants felt the mirror reflection was their right hand in the present study, the cluster in the OTC may be related to the illusory hand ownership.

The EBA is also positioned in the cortical network for visuo-motor interactions; it processes visual information of one’s own body, such as the position, posture, and movements, for actions (Astafiev et al., 2004; Bracci et al., 2012; Orlov et al., 2014; Zimmermann et al., 2018). In the hardness estimation task of the present study, the displacement, speed, and appearance of the strain of the fingers to touch the pad were different between all three conditions, which was detectable by multivariate analysis in the pair-wise comparisons. Furthermore, our previous study showed that participants actually used the visual cues on the finger movements for the hardness estimation along with those on the surface deformation of the pad (Katsuyama et al., 2018). Therefore, the cluster in OTC may also be engaged in processing visual information about finger movements during the hardness estimation task.

### Illusory Ownership and Agency of the Reflection Hand

The result of the hand ownership test indicated that the participants felt as if the reflection of the left hand was the right hand behind the mirror. Illusory ownership toward a reflection of a hand has been previously reported (Longo et al., 2009; Mancini et al., 2011; Takasugi et al., 2011; Hoermann et al., 2012; Jenkinson and Preston, 2015). The present result is consistent with such findings. However, the result of the sense of agency test was somewhat different to that of the illusory hand ownership test. The result indicated that the participants felt that the motion of the hand in the reflection was caused by their own will. However, the answer to the question of whether the hand they intended to move was the left or right hand was biased toward the right, but not significantly. These results indicate that participants felt as if the hand in the reflection was their right hand, but the motion was not necessarily the same as that intended for the right hand. Using the moving rubber hand illusion, a previous study revealed that ownership and agency toward a dummy hand is dissociable (Kalckert and Ehrsson, 2012). In the study, participants observed a dummy hand tapping in synchrony with their hidden hand (the dummy and hidden hands were mechanically connected). When the hidden and dummy hands were moved passively in synchrony by an experimenter, the participants experienced ownership but not agency of the dummy hand. However, when the participants observed the dummy hand moving in synchrony with their hidden hand, the participants experienced agency but not ownership of the dummy hand, if the spatial position of the dummy hand was anatomically incongruent. In the present study, some participants reported that they experienced a peculiar feeling during the scan when they realized that the finger displacements observed in the mirror reflection appeared to differ from those perceived by the proprioceptive sensation. The dissociation of the observed and experienced finger displacements may have been due to the diminished agency of the reflection. Previous studies have revealed that cortical regions in the prefrontal and parietal cortices related to intention and monitoring of actions, such as pre-supplementary motor area (pre-SMA), dorsolateral prefrontal cortex (DLPFC), TPJ, and PPC are also involved in the sense of agency (Tsakiris et al., 2010; Yomogida et al., 2010; Sperduti et al., 2011; Chambon et al., 2013; Kühn et al., 2013). The absence of clusters in these cortical regions in the present study may be due to the imperfections in the agency illusion.

As described earlier, the illusory ownership of dummy hands is encoded in several cortical regions, such as vPM, rTPJ, PPC, and EBA. In the present study, although the participants experienced significant ownership of the hand reflection, no clusters were found in these cortical areas, except for the EBA. One possible reason for this is that the object to which participants experienced the ownership of the right hand was the mirror reflection of the left hand in all three conditions (SFTvm, HRDvm, and MED), making it difficult to detect differences in the pattern of cortical activation between the conditions with respect to the illusory hand ownership. Another reason would be that the illusory hand ownership observed in the present study was not accompanied by a change in the proprioceptive sensation of the hand position. In previous studies, the dummy hand was placed at an anatomically plausible, but apparently different position from that of participant’s real (hidden) hand. After application of synchronous tactile stimulation to the real and dummy hands, the participants felt as though their real hand was in the same position as the dummy hand. The relative displacement between the observed and experienced position of the hands is defined as proprioceptive drift, and has been used as an objective measure of ownership of dummy hands (Botvinick and Cohen, 1998; Holmes and Spence, 2005; Tsakiris and Haggard, 2005). The previous functional imaging studies showing the involvement of vPM, PPC, rTPJ, and EBA in illusory hand ownership employed experimental paradigms including proprioceptive drift (Ehrsson et al., 2004, 2005; Tsakiris et al., 2008; Brozzoli et al., 2012; Limanowski et al., 2014; Limanowski and Blankenburg, 2015). Thus, both illusory ownership and proprioceptive drift may be necessary for activation of specific cortical regions. In contrast, in the present study, the participants were requested to adjust their hands at each side of the mirror such that the reflection of the left hand appeared to be the right hand at the beginning of each run, and the hands were placed at symmetrical positions to the mirror. In such conditions, proprioceptive drift could not be induced. Recent studies have revealed that subjective hand ownership assessed by questionnaires may be dissociable from proprioceptive sensation of the hand position (Holmes et al., 2006; Longo et al., 2008; Rohde et al., 2011; Romano et al., 2015). The exact relationship between illusory hand ownership and the proprioceptive sense of the hand position, and the underlying cortical mechanisms are interesting points for future studies.

## Conclusion

In the present study, we investigated the cortical regions involved in the subjective perception of hardness achieved by integration of visual and haptic information. Participants were required to touch a polyurethane foam pad of constant physical hardness with their right (hidden) hand while observing the mirror reflection of their left hand touching a pad of different hardness, and to estimate the hardness of the pad perceived by the right hand. The behavioral results showed that when they observed the mirror reflection touching the softer or harder pad, they perceived the pad in their right hand as softer or harder, respectively. An fMRI experiment using multivariate analysis suggested that the visual information of finger movements processed in the bilateral OTC may be integrated with haptic input in the left anterior intraparietal sulcus (aIPS), and that the subjective hardness with visual modulation perceived by the right hand may be processed in the cortical network between the left aIPS and parietal operculum (PO).

## Ethics Statement

This study was approved by the local Ethical Committee for the Faculty of Dentistry of the Tokyo Medical and Dental University (D2016-051), Primate Research Institute of Kyoto University (2016-13), Tamagawa University (N27-42), and corresponded to the Human Subjects Guidelines of the Declaration of Helsinki.

## Author Contributions

NK, MT, and KN planned the research project and acquired funding. YK, NU, MT, and NK designed the study. YK, AM, TH, KM, and NK collected the experimental data. YK and NK analyzed the experimental data and prepared the manuscript with supports from NU and KN.

## Conflict of Interest

The authors declare that the research was conducted in the absence of any commercial or financial relationships that could be construed as a potential conflict of interest.

## Footnotes

1 SFT, MED, HRD; SFT, HRD, MED; MED, SFT, HRD; MED, HRD, SFT; HRD, SFT, MED; HRD, MED, SFT.

2 http://www.fil.ion.ucl.ac.uk/spm/

3 Afferents innervating mechanoreceptors that respond to rapid skin indentation but not to steady pressure.

4 Afferents innervating Pacinian corpuscles that are in the deep layers of skin and respond to high frequency vibration (60–400 Hz).

5 Afferents innervating mechanoreceptors in the deep layers of skin that respond to steady skin indentation with a sustained discharge.

## References

[B1] Amaro E. Jr. Barker G. J. (2006). Study design in fMRI: basic principles. *Brain Cogn.* 60 220–232. 10.1016/j.bandc.2005.11.009 16427175

[B2] Arzy S. Thut G. Mohr C. Michel C. M. Blanke O. (2006). Neural basis of embodiment: distinct contributions of temporoparietal junction and extrastriate body area. *J. Neurosci.* 26 8074–8081. 10.1523/JNEUROSCI.0745-06.2006 16885221 PMC6673771

[B3] Astafiev S. V. Stanley C. M. Shulman G. L. Corbetta M. (2004). Extrastriate body area in human occipital cortex responds to the performance of motor actions. *Nat. Neurosci.* 7 542–548. 10.1038/nn1241 15107859

[B4] Avillac M. Ben Hamed S. Duhamel J.-R. (2007). Multisensory integration in the ventral intraparietal area of the macaque monkey. *J. Neurosci.* 27 1922–1932. 10.1523/JNEUROSCI.2646-06.2007 17314288 PMC6673547

[B5] Bekrater-Bodmann R. Foell J. Diers M. Kamping S. Rance M. Kirsch P. (2014). The importance of synchrony and temporal order of visual and tactile input for illusory limb ownership experiences - an FMRI study applying virtual reality. *PLoS One* 9:e87013. 10.1371/journal.pone.0087013 24498012 PMC3909015

[B6] Bensmaia S. (2016). “Texture from touch,” in *Scholarpedia of Touch*, eds Prescott T. Ahissar E. Izhikevich E., (Paris: Atlantis Press), 207–215. 10.2991/978-94-6239-133-8_16

[B7] Bensmaia S. J. Denchev P. V. Dammann J. F. III Craig J. C. Hsiao S. S. (2008). The representation of stimulus orientation in the early stages of somatosensory processing. *J. Neurosci.* 28 776–786. 10.1523/JNEUROSCI.4162-07.2008 18199777 PMC6670339

[B8] Bertamini M. Berselli N. Bode C. Lawson R. Wong L. T. (2011). The rubber hand illusion in a mirror. *Conscious. Cogn.* 20 1108–1119. 10.1016/j.concog.2011.04.006 21536458

[B9] Binkofski F. Buccino G. Posse S. Seitz R. J. Rizzolatti G. Freund H. (1999). A fronto-parietal circuit for object manipulation in man: evidence from an fMRI-study. *Eur. J. Neurosci.* 11 3276–3286. 10.1046/j.1460-9568.1999.00753.x 10510191

[B10] Björnsdotter M. Gordon I. Pelphrey K. A. Olausson H. Kaiser M. D. (2014). Development of brain mechanisms for processing affective touch. *Front. Behav. Neurosci.* 8:24. 10.3389/fnbeh.2014.00024 24550800 PMC3912430

[B11] Blankenburg F. Ruff C. C. Deichmann R. Rees G. Driver J. (2006). The cutaneous rabbit illusion affects human primary sensory cortex somatotopically. *PLoS Biol.* 4:e69. 10.1371/journal.pbio.0040069 16494530 PMC1382015

[B12] Botvinick M. Cohen J. (1998). Rubber hands ‘feel’ touch that eyes see. *Nature* 391:756. 10.1038/35784 9486643

[B13] Bowsher D. Brooks J. Enevoldson P. (2004). Central representation of somatic sensations in the parietal operculum (SII) and insula. *Eur. Neurol.* 52 211–225. 10.1159/000082038 15539775

[B14] Bracci S. Cavina-Pratesi C. Ietswaart M. Caramazza A. Peelen M. V. (2012). Closely overlapping responses to tools and hands in left lateral occipitotemporal cortex. *J. Neurophysiol.* 107 1443–1456. 10.1152/jn.00619.2011 22131379

[B15] Brozzoli C. Gentile G. Ehrsson H. H. (2012). That’s near my hand! parietal and premotor coding of hand-centered space contributes to localization and self-attribution of the hand. *J. Neurosci.* 32 14573–14582. 10.1523/JNEUROSCI.2660-12.2012 23077043 PMC6621451

[B16] Burton H. Abend N. S. MacLeod A. M. Sinclair R. J. Snyder A. Z. Raichle M. E. (1999). Tactile attention tasks enhance activation in somatosensory regions of parietal cortex: a positron emission tomography study. *Cereb. Cortex* 9 662–674. 10.1093/cercor/9.7.662 10554989

[B17] Burton H. Sinclair R. J. McLaren D. G. (2008). Cortical network for vibrotactile attention: a fMRI study. *Hum. Brain Mapp.* 29 207–221. 10.1002/hbm.20384 17390318 PMC2593407

[B18] Carmichael S. T. Price J. L. (1995). Sensory and premotor connections of the orbital and medial prefrontal cortex of macaque monkeys. *J. Comp. Neurol.* 363 642–664. 10.1002/cne.903630409 8847422

[B19] Caselli R. J. (1991). Rediscovering tactile agnosia. *Mayo Clin. Proc.* 66 129–142. 10.1016/s0025-6196(12)60484-4 1994134

[B20] Cellini C. Kaim L. Drewing K. (2013). Visual and haptic integration in the estimation of softness of deformable objects. *Iperception* 4 516–531. 10.1068/i0598 25165510 PMC4129386

[B21] Chambon V. Wenke D. Fleming S. M. Prinz W. Haggard P. (2013). An online neural substrate for sense of agency. *Cereb. Cortex* 23 1031–1037. 10.1093/cercor/bhs059 22510529

[B22] Chang C. C. Lin C. J. (2011). LIBSVM: a library for support vector machines. *ACM Trans. Intell. Syst. Technol.* 2 1–27. 10.1145/1961189.1961199

[B23] Chen L. M. Friedman R. M. Roe A. W. (2003). Optical imaging of a tactile illusion in area 3b of the primary somatosensory cortex. *Science* 302 881–885. 10.1126/science.1087846 14500850

[B24] Colby C. L. Duhamel J.-R. (1991). Heterogeneity of extrastriate visual areas and multiple parietal areas in the macaque monkey. *Neuropsychologia* 29 517–537. 10.1016/0028-3932(91)90008-V 1944859

[B25] Costantini M. Urgesi C. Galati G. Romani G. L. Aglioti S. M. (2011). Haptic perception and body representation in lateral and medial occipito-temporal cortices. *Neuropsychologia* 49 821–829. 10.1016/j.neuropsychologia.2011.01.034 21316376

[B26] Cusick C. G. Wall J. T. Felleman D. J. Kaas J. H. (1989). Somatotopic organization of the lateral sulcus of owl monkeys: area 3b, S-II, and a ventral somatosensory area. *J. Comp. Neurol.* 282 169–190. 10.1002/cne.902820203 2496153

[B27] Delhaye B. P. Long K. H. Bensmaia S. J. (2018). Neural basis of touch and proprioception in primate cortex. *Compr. Physiol.* 8 1575–1602. 10.1002/cphy.c170033 30215864 PMC6330897

[B28] Disbrow E. Roberts T. Krubitzer L. (2000). Somatotopic organization of cortical fields in the lateral sulcus of homo sapiens: evidence for SII and PV. *J. Comp. Neurol.* 418 1–21. 10.1002/(sici)1096-9861(20000228)418:1<1::aid-cne1>3.0.co;2-p 10701752

[B29] Disbrow E. Roberts T. Poeppel D. Krubitzer L. (2001). Evidence for interhemispheric processing of inputs from the hands in human S2 and PV. *J. Neurophysiol.* 85 2236–2244. 10.1152/jn.2001.85.5.2236 11353038

[B30] Downing P. E. Jiang Y. Shuman M. Kanwisher N. (2001). A cortical area selective for visual processing of the human body. *Science* 293 2470–2473. 10.1126/science.1063414 11577239

[B31] Duhamel J.-R. Colby C. L. Goldberg M. E. (1998). Ventral intraparietal area of the macaque: congruent visual and somatic response properties. *J. Neurophysiol.* 79 126–136. 10.1152/jn.1998.79.1.126 9425183

[B32] Ehrsson H. H. Holmes N. P. Passingham R. E. (2005). Touching a rubber hand: feeling of body ownership is associated with activity in multisensory brain areas. *J. Neurosci.* 25 10564–10573. 10.1523/JNEUROSCI.0800-05.2005 16280594 PMC1395356

[B33] Ehrsson H. H. Spence C. Passingham R. E. (2004). That’s my hand! Activity in premotor cortex reflects feeling of ownership of a limb. *Science* 305 875–877. 10.1126/science.1097011 15232072

[B34] Eickhoff S. B. Grefkes C. Zilles K. Fink G. R. (2007). The somatotopic organization of cytoarchitectonic areas on the human parietal operculum. *Cereb. Cortex* 17 1800–1811. 10.1093/cercor/bhl090 17032710

[B35] Eickhoff S. B. Jbabdi S. Caspers S. Laird A. R. Fox P. T. Zilles K. (2010). Anatomical and functional connectivity of cytoarchitectonic areas within the human parietal operculum. *J. Neurosci.* 30 6409–6421. 10.1523/JNEUROSCI.5664-09.2010 20445067 PMC4791040

[B36] Eickhoff S. B. Schleicher A. Zilles K. Amunts K. (2006). The human parietal operculum. I. Cytoarchitectonic mapping of subdivisions. *Cereb. Cortex* 16 254–267. 10.1093/cercor/bhi105 15888607

[B37] Eickhoff S. B. Stephan K. E. Mohlberg H. Grefkes C. Fink G. R. Amunts K. (2005). A new SPM toolbox for combining probabilistic cytoarchitectonic maps and functional imaging data. *Neuroimage* 25 1325–1335. 10.1016/j.neuroimage.2004.12.034 15850749

[B38] Ferri S. Kolster H. Jastorff J. Orban G. A. (2013). The overlap of the EBA and the MT/V5 cluster. *Neuroimage* 66 412–425. 10.1016/j.neuroimage.2012.10.060 23108274

[B39] Fitzgerald P. J. Lane J. W. Thakur P. H. Hsiao S. S. (2006a). Receptive field properties of the macaque second somatosensory cortex: representation of orientation on different finger pads. *J. Neurosci.* 26 6473–6484. 10.1523/JNEUROSCI.5057-05.2006 16775135 PMC1839049

[B40] Fitzgerald P. J. Lane J. W. Thakur P. H. Hsiao S. S. (2006b). Receptive field (RF) properties of the macaque second somatosensory cortex: RF size, shape, and somatotopic organization. *J. Neurosci.* 26 6485–6495. 10.1523/JNEUROSCI.5061-05.2006 16775136 PMC1800881

[B41] Friedman D. P. Murray E. A. O’Neill J. B. Mishkin M. (1986). Cortical connections of the somatosensory fields of the lateral sulcus of macaques: evidence for a corticolimbic pathway for touch. *J. Comp. Neurol.* 252 323–347. 10.1002/cne.902520304 3793980

[B42] Friedman R. M. Hester K. D. Green B. G. LaMotte R. H. (2008). Magnitude estimation of softness. *Exp. Brain Res.* 191 133–142. 10.1007/s00221-008-1507-5 18679665 PMC2574806

[B43] Friston K. J. Price C. J. Fletcher P. Moore C. Frackowiak R. S. Dolan R. J. (1996). The trouble with cognitive subtraction. *Neuroimage* 4 97–104. 10.1006/nimg.1996.0033 9345501

[B44] Gentile G. Petkova V. I. Ehrsson H. H. (2011). Integration of visual and tactile signals from the hand in the human brain: an FMRI study. *J. Neurophysiol.* 105 910–922. 10.1152/jn.00840.2010 21148091 PMC3059180

[B45] Gescheider G. A. (1997). *Psychophysics: The Fundamentals*, 3rd Edn New Jersey, NJ: Lawrence Erlbaum Associates. Publishers.

[B46] Grefkes C. Weiss P. H. Zilles K. Fink G. R. (2002). Crossmodal processing of object features in human anterior intraparietal cortex. *Neuron* 35 173–184. 10.1016/s0896-6273(02)00741-9 12123617

[B47] Guipponi O. Wardak C. Ibarrola D. Comte J. C. Sappey-Marinier D. Pinede S. (2013). Multimodal convergence within the intraparietal sulcus of the macaque monkey. *J. Neurosci.* 33 4128–4139. 10.1523/JNEUROSCI.1421-12.2013 23447621 PMC6619317

[B48] Harrington T. Merzenich M. M. (1970). Neural coding in the sense of touch: human sensations of skin indentation compared with the responses of slowly adapting mechanoreceptive afferents innvervating the hairy skin of monkeys. *Exp. Brain Res.* 10 251–264. 4985999 10.1007/BF00235049

[B49] Hebart M. N. Görgen K. Haynes J. D. (2015). The decoding toolbox (TDT): a versatile software package for multivariate analyses of functional imaging data. *Front. Neuroinform.* 8:88. 10.3389/fninf.2014.00088 25610393 PMC4285115

[B50] Hernández-Pérez R. Cuaya L. V. Rojas-Hortelano E. Reyes-Aguilar A. Concha L. de Lafuente V. (2017). Tactile object categories can be decoded from the parietal and lateral-occipital cortices. *Neuroscience* 352 226–235. 10.1016/j.neuroscience.2017.03.038 28377175

[B51] Hinkley L. B. Krubitzer L. A. Nagarajan S. S. Disbrow E. A. (2007). Sensorimotor integration in S2, PV, and parietal rostroventral areas of the human sylvian fissure. *J. Neurophysiol.* 97 1288–1297. 10.1152/jn.00733.2006 17122318 PMC4060608

[B52] Hinkley L. B. Krubitzer L. A. Padberg J. Disbrow E. A. (2009). Visual-manual exploration and posterior parietal cortex in humans. *J. Neurophysiol.* 102 3433–3446. 10.1152/jn.90785.2008 19812283 PMC2804435

[B53] Hoermann S. Franz E. A. Regenbrecht H. (2012). Referred sensations elicited by video-mediated mirroring of hands. *PLoS One* 7:e50942. 10.1371/journal.pone.0050942 23272080 PMC3525577

[B54] Holmes N. P. Snijders H. J. Spence C. (2006). Reaching with alien limbs: visual exposure to prosthetic hands in a mirror biases proprioception without accompanying illusions of ownership. *Percept. Psychophys.* 68 685–701. 10.3758/bf03208768 16933431 PMC1564193

[B55] Holmes N. P. Spence C. (2005). Visual bias of unseen hand position with a mirror: spatial and temporal factors. *Exp. Brain Res.* 166 489–497. 10.1007/s00221-005-2389-4 16032401 PMC1343466

[B56] Hötting K. Friedrich C. K. Röder B. (2009). Neural correlates of cross-modally induced changes in tactile awareness. *J. Cogn. Neurosci.* 21 2445–2461. 10.1162/jocn.2008.21177 19199403

[B57] Huang R. S. Sereno M. I. (2018). Multisensory and sensorimotor maps. *Handb. Clin. Neurol.* 151 141–161. 10.1016/B978-0-444-63622-5.00007-3 29519456

[B58] Ionta S. Heydrich L. Lenggenhager B. Mouthon M. Fornari E. Chapuis D. (2011). Multisensory mechanisms in temporo-parietal cortex support self-location and first-person perspective. *Neuron* 70 363–374. 10.1016/j.neuron.2011.03.009 21521620

[B59] Iwamura Y. Tanaka M. Hikosaka O. (1980). Overlapping representation of fingers in the somatosensory cortex (area 2) of the conscious monkey. *Brain Res.* 197 516–520. 10.1016/0006-8993(80)91139-7 7407570

[B60] Iwamura Y. Tanaka M. Sakamoto M. Hikosaka O. (1985a). “Comparison of the hand and finger representation in area 3, 1, and 2 of the monkey somatosensory cortex,” in *Development, Organization, and processing in Somatosensory Pathways*, ed. Rowe W. D., (New York: Liss), 239–245.

[B61] Iwamura Y. Tanaka M. Sakamoto M. Hikosaka O. (1985b). Vertical neuronal arrays in the postcentral gyrus signaling active touch: a receptive field study in the conscious monkey. *Exp. Brain Res.* 58 412–420. 10.1007/bf00235322 3996504

[B62] Jenkinson P. M. Preston C. (2015). New reflections on agency and body ownership: the moving rubber hand illusion in the mirror. *Conscious. Cogn.* 33 432–442. 10.1016/j.concog.2015.02.020 25792444

[B63] Joanne Jao R. James T. W. Harman James K. (2014). Multisensory convergence of visual and haptic object preference across development. *Neuropsychologia* 56 381–392. 10.1016/j.neuropsychologia.2014.02.009 24560914 PMC4020146

[B64] Johansson R. S. Flanagan J. R. (2009). Coding and use of tactile signals from the fingertips in object manipulation tasks. *Nat. Rev. Neurosci.* 10 345–359. 10.1038/nrn2621 19352402

[B65] Kalckert A. Ehrsson H. H. (2012). Moving a rubber hand that feels like your own: a dissociation of ownership and agency. *Front. Hum. Neurosci.* 6:40. 10.3389/fnhum.2012.00040 22435056 PMC3303087

[B66] Kalckert A. Ehrsson H. H. (2014). The spatial distance rule in the moving and classical rubber hand illusions. *Conscious. Cogn.* 30 118–132. 10.1016/j.concog.2014.08.022 25286241

[B67] Kassuba T. Klinge C. Holig C. Roder B. Siebner H. R. (2013). Vision holds a greater share in visuo-haptic object recognition than touch. *Neuroimage* 65 59–68. 10.1016/j.neuroimage.2012.09.054 23032487

[B68] Katsuyama N. Kikuchi-Tachi E. Usui N. Yoshizawa H. Saito A. Taira M. (2018). Effect of visual information on active touch during mirror visual feedback. *Front. Hum. Neurosci.* 12:424. 10.3389/fnhum.2018.00424 30405378 PMC6200852

[B69] Keysers C. Wicker B. Gazzola V. Anton J. L. Fogassi L. Gallese V. (2004). A touching sight: SII/PV activation during the observation and experience of touch. *Neuron* 42 335–346. 10.1016/S0896-6273(04)00156-4 15091347

[B70] Kim J. Yeon J. Ryu J. Park J. Y. Chung S. C. Kim S. P. (2017). Neural activity patterns in the human brain reflect tactile stickiness perception. *Front. Hum. Neurosci.* 11:445. 10.3389/fnhum.2017.00445 28936171 PMC5595153

[B71] Kitada R. Kito T. Saito D. N. Kochiyama T. Matsumura M. Sadato N. (2006). Multisensory activation of the intraparietal area when classifying grating orientation: a functional magnetic resonance imaging study. *J. Neurosci.* 26 7491–7501. 10.1523/JNEUROSCI.0822-06.2006 16837597 PMC6674180

[B72] Kitada R. Sasaki A. T. Okamoto Y. Kochiyama T. Sadato N. (2014a). Role of the precuneus in the detection of incongruency between tactile and visual texture information: a functional MRI study. *Neuropsychologia* 64 252–262. 10.1016/j.neuropsychologia.2014.09.028 25281887

[B73] Kitada R. Yoshihara K. Sasaki A. T. Hashiguchi M. Kochiyama T. Sadato N. (2014b). The brain network underlying the recognition of hand gestures in the blind: the supramodal role of the extrastriate body area. *J. Neurosci.* 34 10096–10108. 10.1523/JNEUROSCI.0500-14.2014 25057211 PMC6608300

[B74] Klatzky R. L. Lederman S. J. Reed C. (1987). There’s more to touch than meets the eye: the salience of object attributes for haptics with and without vision. *J. Exp. Psychol. Gen.* 116 356–369. 10.1037//0096-3445.116.4.356

[B75] Kokkinara E. Slater M. (2014). Measuring the effects through time of the influence of visuomotor and visuotactile synchronous stimulation on a virtual body ownership illusion. *Perception* 43 43–58. 10.1068/p7545 24689131

[B76] Kriegeskorte N. Goebel R. Bandettini P. (2006). Information-based functional brain mapping. *Proc. Natl. Acad. Sci. U.S.A.* 103 3863–3868. 10.1073/pnas.0600244103 16537458 PMC1383651

[B77] Krubitzer L. Clarey J. Tweedale R. Elston G. Calford M. (1995). A redefinition of somatosensory areas in the lateral sulcus of macaque monkeys. *J. Neurosci.* 15 3821–3839. 10.1523/JNEUROSCI.15-05-03821.1995 7751949 PMC6578217

[B78] Kühn S. Brass M. Haggard P. (2013). Feeling in control: neural correlates of experience of agency. *Cortex* 49 1935–1942. 10.1016/j.cortex.2012.09.002 23067726

[B79] Kurth R. Villringer K. Curio G. Wolf K. J. Krause T. Repenthin J. (2000). fMRI shows multiple somatotopic digit representations in human primary somatosensory cortex. *Neuroreport* 11 1487–1491. 10.1097/00001756-200005150-00026 10841363

[B80] Kuschel M. Di Luca M. Buss M. Klatzky R. L. (2010). Combination and integration in the perception of visual-haptic compliance information. *IEEE Trans. Haptics* 3 234–244. 10.1109/TOH.2010.9 27788109

[B81] Leib R. Mawase F. Karniel A. Donchin O. Rothwell J. Nisky I. (2016). Stimulation of PPC affects the mapping between motion and force signals for stiffness perception but not motion control. *J. Neurosci.* 36 10545–10559. 10.1523/JNEUROSCI.1178-16.2016 27733607 PMC5059428

[B82] Limanowski J. Blankenburg F. (2015). Network activity underlying the illusory self-attribution of a dummy arm. *Hum. Brain Mapp.* 36 2284–2304. 10.1002/hbm.22770 25708317 PMC6869824

[B83] Limanowski J. Lutti A. Blankenburg F. (2014). The extrastriate body area is involved in illusory limb ownership. *Neuroimage* 86 514–524. 10.1016/j.neuroimage.2013.10.035 24185016

[B84] Lin Y. Y. Forss N. (2002). Functional characterization of human second somatosensory cortex by magnetoencephalography. *Behav. Brain Res.* 135 141–145. 10.1016/S0166-4328(02)00143-2 12356444

[B85] Longo M. R. Betti V. Aglioti S. M. Haggard P. (2009). Visually induced analgesia: seeing the body reduces pain. *J. Neurosci.* 29 12125–12130. 10.1523/JNEUROSCI.3072-09.2009 19793970 PMC6666129

[B86] Longo M. R. Schuur F. Kammers M. P. Tsakiris M. Haggard P. (2008). What is embodiment? A psychometric approach. *Cognition* 107 978–998. 10.1016/j.cognition.2007.12.004 18262508

[B87] Lüders H. Lesser R. P. Dinner D. S. Hahn J. F. Salanga V. Morris H. H. (1985). The second sensory area in humans: evoked potential and electrical stimulation studies. *Ann. Neurol.* 17 177–184. 10.1002/ana.410170212 3977299

[B88] Ma K. Hommel B. (2015). Body-ownership for actively operated non-corporeal objects. *Conscious. Cogn.* 36 75–86. 10.1016/j.concog.2015.06.003 26094223

[B89] Macaluso E. Driver J. (2001). Spatial attention and crossmodal interactions between vision and touch. *Neuropsychologia* 39 1304–1316. 10.1016/S0028-3932(01)00119-1 11566313

[B90] Mãlîia M. D. Donos C. Barborica A. Popa I. Ciurea J. Cinatti S. (2018). Functional mapping and effective connectivity of the human operculum. *Cortex* 109 303–321. 10.1016/j.cortex.2018.08.024 30414541

[B91] Mancini F. Longo M. R. Kammers M. P. Haggard P. (2011). Visual distortion of body size modulates pain perception. *Psychol. Sci.* 22 325–330. 10.1177/0956797611398496 21303990

[B92] Matelli M. Camarda R. Glickstein M. Rizzolatti G. (1986). Afferent and efferent projections of the inferior area 6 in the macaque monkey. *J. Comp. Neurol.* 251 281–298. 10.1002/cne.902510302 3021823

[B93] Matthys K. Smits M. Van der Geest J. N. Van der Lugt A. Seurinck R. Stam H. J. (2009). Mirror-induced visual illusion of hand movements: a functional magnetic resonance imaging study. *Arch. Phys. Med. Rehabil.* 90 675–681. 10.1016/j.apmr.2008.09.571 19345786

[B94] Mazzola L. Faillenot I. Barral F. G. Mauguière F. Peyron R. (2012). Spatial segregation of somato-sensory and pain activations in the human operculo-insular cortex. *Neuroimage* 60 409–418. 10.1016/j.neuroimage.2011.12.072 22245639

[B95] Morrison I. (2016). ALE meta-analysis reveals dissociable networks for affective and discriminative aspects of touch. *Hum. Brain Mapp.* 37 1308–1320. 10.1002/hbm.23103 26873519 PMC5066805

[B96] Mountcastle V. B. Talbot W. H. Kornhuber H. H. (1966). “The neural transformation of mechanical stimuli delivered to the monkey’s hand,” in *Ciba Foundation Symposium: Touch, Heat, and Pain*, eds de Reuck A. V. S. Knight J., (London: Churchill), 325–351. 10.1002/9780470715338.ch19

[B97] Mufson E. J. Mesulam M. M. Pandya D. N. (1981). Insular interconnections with the amygdala in the rhesus monkey. *Neuroscience* 6 1231–1248. 10.1016/0306-4522(81)90184-6 6167896

[B98] Muniak M. A. Ray S. Hsiao S. S. Dammann J. F. Bensmaia S. J. (2007). The neural coding of stimulus intensity: linking the population response of mechanoreceptive afferents with psychophysical behavior. *J. Neurosci.* 27 11687–11699. 10.1523/JNEUROSCI.1486-07.2007 17959811 PMC6673240

[B99] Naito E. Ehrsson H. H. (2006). Somatic sensation of hand-object interactive movement is associated with activity in the left inferior parietal cortex. *J. Neurosci.* 26 3783–3790. 10.1523/JNEUROSCI.4835-05.2006 16597731 PMC6674143

[B100] Newman S. D. Klatzky R. L. Lederman S. J. Just M. A. (2005). Imagining material versus geometric properties of objects: an fMRI study. *Brain Res. Cogn. Brain Res.* 23 235–246. 10.1016/j.cogbrainres.2004.10.020 15820631

[B101] Nichols T. Brett M. Andersson J. Wager T. Poline J. B. (2005). Valid conjunction inference with the minimum statistic. *Neuroimage* 25 653–660. 10.1016/j.neuroimage.2004.12.005 15808966

[B102] Okamoto S. Nagano H. Yamada Y. (2013). Psychophysical dimensions of tactile perception of textures. *IEEE Trans. Haptics* 6 81–93. 10.1109/TOH.2012.32 24808270

[B103] Oldfield R. C. (1971). The assessment and analysis of handedness: the edinburgh inventory. *Neuropsychologia* 9 97–113. 10.1016/0028-3932(71)90067-4 5146491

[B104] Orlov T. Makin T. R. Zohary E. (2010). Topographic representation of the human body in the occipitotemporal cortex. *Neuron* 68 586–600. 10.1016/j.neuron.2010.09.032 21040856

[B105] Orlov T. Porat Y. Makin T. R. Zohary E. (2014). Hands in motion: an upper-limb-selective area in the occipitotemporal cortex shows sensitivity to viewed hand kinematics. *J. Neurosci.* 34 4882–4895. 10.1523/JNEUROSCI.3352-13.2014 24695707 PMC6802726

[B106] Pandya D. N. Kuypers H. G. (1969). Cortico-cortical connections in the rhesus monkey. *Brain Res.* 13 13–36. 10.1016/0006-8993(69)90141-3 4185124

[B107] Pasalar S. Ro T. Beauchamp M. S. (2010). TMS of posterior parietal cortex disrupts visual tactile multisensory integration. *Eur. J. Neurosci.* 31 1783–1790. 10.1111/j.1460-9568.2010.07193.x 20584182 PMC2994715

[B108] Penfield W. Jasper H. (1954). *Epilepsy and The Functional Anatomy of The Human Brain.* Boston: Little Brown.

[B109] Petersen S. E. Fox P. T. Posner M. I. Mintun M. Raichle M. E. (1989). Positron emission tomographic studies of the processing of singe words. *J. Cogn. Neurosci.* 1 153–170. 10.1162/jocn.1989.1.2.153 23968463

[B110] Pietrini P. Furey M. L. Ricciardi E. Gobbini M. I. Wu W. H. C. Cohen L. (2004). Beyond sensory images: object-based representation in the human ventral pathway. *Proc. Natl. Acad. Sci. U.S.A.* 101 5658–5663. 10.1073/pnas.0400707101 15064396 PMC397466

[B111] Podrebarac S. K. Goodale M. A. Snow J. C. (2014). Are visual texture-selective areas recruited during haptic texture discrimination? *Neuroimage* 94 129–137. 10.1016/j.neuroimage.2014.03.013 24650604

[B112] Punpongsanon P. Iwai D. Sato K. (2015). SoftAR: visually manipulating haptic softness perception in spatial augmented reality. *IEEE Trans. Vis. Comput. Graph.* 21 1279–1288. 10.1109/TVCG.2015.2459792 26340774

[B113] Roberts R. D. (2013). Roughness perception across the hands. *Atten. Percept. Psychophys.* 75 1306–1317. 10.3758/s13414-013-0465-6 23653412

[B114] Rohde M. Di Luca M. Ernst M. O. (2011). The rubber hand illusion: feeling of ownership and proprioceptive drift do not go hand in hand. *PLoS One* 6:e21659. 10.1371/journal.pone.0021659 21738756 PMC3125296

[B115] Romano D. Caffa E. Hernandez-Arieta A. Brugger P. Maravita A. (2015). The robot hand illusion: inducing proprioceptive drift through visuo-motor congruency. *Neuropsychologia* 70 414–420. 10.1016/j.neuropsychologia.2014.10.033 25446964

[B116] Ruben J. Schwiemann J. Deuchert M. Meyer R. Krause T. Curio G. (2001). Somatotopic organization of human secondary somatosensory cortex. *Cereb. Cortex* 11 463–473. 10.1093/cercor/11.5.463 11313298

[B117] Sanchez-Vives M. V. Spanlang B. Frisoli A. Bergamasco M. Slater M. (2010). Virtual hand illusion induced by visuomotor correlations. *PLoS One* 5:e10381. 10.1371/journal.pone.0010381 20454463 PMC2861624

[B118] Schaefer M. Noennig N. Heinze H. J. Rotte M. (2006). Fooling your feelings: artificially induced referred sensations are linked to a modulation of the primary somatosensory cortex. *Neuroimage* 29 67–73. 10.1016/j.neuroimage.2005.07.001 16054839

[B119] Schmidt T. T. Ostwald D. Blankenburg F. (2014). Imaging tactile imagery: changes in brain connectivity support perceptual grounding of mental images in primary sensory cortices. *Neuroimage* 98 216–224. 10.1016/j.neuroimage.2014.05.014 24836010

[B120] Servos P. Lederman S. Wilson D. Gati J. (2001). fMRI-derived cortical maps for haptic shape, texture, and hardness. *Brain Res. Cogn. Brain Res.* 12 307–313. 10.1016/S0926-6410(01)00041-6 11587899

[B121] Shokur S. O’Doherty J. E. Winans J. A. Bleuler H. Lebedev M. A. Nicolelis M. A. (2013). Expanding the primate body schema in sensorimotor cortex by virtual touches of an avatar. *Proc. Natl. Acad. Sci. U.S.A.* 110 15121–15126. 10.1073/pnas.1308459110 23980141 PMC3773736

[B122] Sinclair R. J. Burton H. (1993). Neuronal activity in the second somatosensory cortex of monkeys (*Macaca mulatta*) during active touch of gratings. *J. Neurophysiol.* 70 331–350. 10.1152/jn.1993.70.1.331 8360718

[B123] Sperduti M. Delaveau P. Fossati P. Nadel J. (2011). Different brain structures related to self- and external-agency attribution: a brief review and meta-analysis. *Brain Struct. Funct.* 216 151–157. 10.1007/s00429-010-0298-1 21212978

[B124] Spiridon M. Fischl B. Kanwisher N. (2006). Location and spatial profile of category-specific regions in human extrastriate cortex. *Hum. Brain Mapp.* 27 77–89. 10.1002/hbm.20169 15966002 PMC3264054

[B125] Srinivasan M. A. LaMotte R. H. (1995). Tactual discrimination of softness. *J. Neurophysiol.* 73 88–101. 10.1152/jn.1995.73.1.88 7714593

[B126] Srinivasan M. A. LaMotte R. H. (1996). “Tactual discrimination of softness: Abilities and mechanisms,” in *Somesthesis and the Neurobiology of the Somatosensory Cortex*, eds Franzen O. Johansson R. Terenius L., (Birkhäuser: Basel), 123–135. 10.1007/978-3-0348-9016-8_11

[B127] Stančák A. Polácek H. Vrána J. Rachmanová R. Hoechstetter K. Tintra J. (2005). EEG source analysis and fMRI reveal two electrical sources in the fronto-parietal operculum during subepidermal finger stimulation. *Neuroimage* 25 8–20. 10.1016/j.neuroimage.2004.10.025 15734339

[B128] Stilla R. Sathian K. (2008). Selective visuo-haptic processing of shape and texture. *Hum. Brain Mapp.* 29 1123–1138. 10.1002/hbm.20456 17924535 PMC3060058

[B129] Takasugi J. Matsuzawa D. Murayama T. Nakazawa K. Numata K. Shimizu E. (2011). Referred sensations induced by a mirror box in healthy subjects. *Psychol. Res.* 75 54–60. 10.1007/s00426-010-0287-2 20505951

[B130] Talairach J. Tournoux P. (1988). *Co-Planar Stereotaxic atlas of the Human Brain: 3-Dimensional Proportional System: An Approach to Cerebral Imaging.* New York, NY: Thieme.

[B131] Tamè L. Braun C. Lingnau A. Schwarzbach J. Demarchi G. Li Hegner Y. (2012). The contribution of primary and secondary somatosensory cortices to the representation of body parts and body sides: an fMRI adaptation study. *J. Cogn. Neurosci.* 24 2306–2320. 10.1162/jocn_a_00272 22849401

[B132] Taoka M. Toda T. Hihara S. Tanaka M. Iriki A. Iwamura Y. (2016). A systematic analysis of neurons with large somatosensory receptive fields covering multiple body regions in the secondary somatosensory area of macaque monkeys. *J. Neurophysiol.* 116 2152–2162. 10.1152/jn.00241.2016 27559139 PMC5102307

[B133] Tsakiris M. Costantini M. Haggard P. (2008). The role of the right temporo-parietal junction in maintaining a coherent sense of one’s body. *Neuropsychologia* 46 3014–3018. 10.1016/j.neuropsychologia.2008.06.004 18601939

[B134] Tsakiris M. Haggard P. (2005). The rubber hand illusion revisited: visuotactile integration and self-attribution. *J. Exp. Psychol. Hum. Percept. Perform.* 31 80–91. 10.1037/0096-1523.31.1.80 15709864

[B135] Tsakiris M. Longo M. R. Haggard P. (2010). Having a body versus moving your body: neural signatures of agency and body-ownership. *Neuropsychologia* 48 2740–2749. 10.1016/j.neuropsychologia.2010.05.021 20510255

[B136] Tsakiris M. Prabhu G. Haggard P. (2006). Having a body versus moving your body: how agency structures body-ownership. *Conscious. Cogn.* 15 423–432. 10.1016/j.concog.2005.09.004 16343947

[B137] Yeon J. Kim J. Ryu J. Park J. Y. Chung S. C. Kim S. P. (2017). Human brain activity related to the tactile perception of stickiness. *Front. Hum. Neurosci.* 11:8. 10.3389/fnhum.2017.00008 28163677 PMC5247468

[B138] Yomogida Y. Sugiura M. Sassa Y. Wakusawa K. Sekiguchi A. Fukushima A. (2010). The neural basis of agency: an fMRI study. *Neuroimage* 50 198–207. 10.1016/j.neuroimage.2009.12.054 20026225

[B139] Zimmermann M. Mars R. B. de Lange F. P. Toni I. Verhagen L. (2018). Is the extrastriate body area part of the dorsal visuomotor stream? *Brain Struct. Funct.* 223 31–46. 10.1007/s00429-017-1469-0 28702735 PMC5772142

