# Supplementary material for: Cortical Regions Encoding Hardness Perception Modulated by Visual Information Identified by Functional Magnetic Resonance Imaging With Multivoxel Pattern Analysis
Source: Front Syst Neurosci. 2019 Oct 1;13:52. doi: 10.3389/fnsys.2019.00052 (PMC6779815; doi:10.3389/fnsys.2019.00052)
Supplement: Supplementary file 1 [file Table_1.DOCX]

**Supplementary Table 1**

Modulation ratio of all runs and participants for conditions SFT and HRD. Participants are listed in descending order of the modulation ratio averaged across conditions and runs.

|  | | Condition | Run | | | | | | | | mean rate |
| --- | --- | --- | --- | --- | --- | --- | --- | --- | --- | --- | --- |
|  |  |  | 1 | 2 | 3 | 4 | 5 | 6 | 7 | 8 |  |
| Participant | 11 | SFT | 100.0 | 100.0 | 100.0 | 100.0 | 100.0 | 100.0 | 100.0 | 100.0 | 100.0 |
|  |  | HRD | 100.0 | 87.5 | 100.0 | 100.0 | 87.5 | 87.5 | 100.0 | 100.0 | 95.3 |
|  | 17 | SFT | 75.0 | 100.0 | 87.5 | 87.5 | 100.0 | 75.0 | 87.5 | 100.0 | 89.1 |
|  |  | HRD | 100.0 | 100.0 | 100.0 | 100.0 | 100.0 | 100.0 | 100.0 | 100.0 | 100.0 |
|  | 4 | SFT | 62.5 | 87.5 | 100.0 | 100.0 | 100.0 | 100.0 | 100.0 | 100.0 | 93.8 |
|  |  | HRD | 87.5 | 87.5 | 100.0 | 75.0 | 100.0 | 87.5 | 100.0 | 100.0 | 92.2 |
|  | 15 | SFT | 75.0 | 87.5 | 87.5 | 87.5 | 100.0 | 87.5 | 100.0 | 75.0 | 87.5 |
|  |  | HRD | 100.0 | 100.0 | 100.0 | 87.5 | 100.0 | 87.5 | 100.0 | 100.0 | 96.9 |
|  | 3 | SFT | 62.5 | 87.5 | 100.0 | 100.0 | 100.0 | 100.0 | 75.0 | 75.0 | 87.5 |
|  |  | HRD | 75.0 | 100.0 | 75.0 | 100.0 | 100.0 | 100.0 | 100.0 | 87.5 | 92.2 |
|  | 7 | SFT | 25.0 | 100.0 | 87.5 | 87.5 | 75.0 | 87.5 | 87.5 | 100.0 | 81.3 |
|  |  | HRD | 75.0 | 75.0 | 100.0 | 100.0 | 87.5 | 100.0 | 87.5 | 100.0 | 90.6 |
|  | 16 | SFT | 62.5 | 87.5 | 87.5 | 87.5 | 87.5 | 75.0 | 100.0 | 50.0 | 79.7 |
|  |  | HRD | 100.0 | 87.5 | 75.0 | 75.0 | 100.0 | 87.5 | 75.0 | 62.5 | 82.8 |
|  | 1 | SFT | 87.5 | 100.0 | 100.0 | 100.0 | 87.5 | 100.0 | 100.0 | 100.0 | 96.9 |
|  |  | HRD | 100.0 | 87.5 | 87.5 | 37.5 | 25.0 | 50.0 | 50.0 | 50.0 | 60.9 |
|  | 9 | SFT | 62.5 | 87.5 | 75.0 | 87.5 | 75.0 | 62.5 | 37.5 | 50.0 | 67.2 |
|  |  | HRD | 75.0 | 100.0 | 87.5 | 100.0 | 75.0 | 100.0 | 100.0 | 87.5 | 90.6 |
|  | 5 | SFT | 100.0 | 75.0 | 75.0 | 100.0 | 100.0 | 87.5 | 87.5 | 87.5 | 89.1 |
|  |  | HRD | 87.5 | 87.5 | 87.5 | 62.5 | 50.0 | 50.0 | 50.0 | 50.0 | 65.6 |
|  | 22 | SFT | 37.5 | 75.0 | 75.0 | 50.0 | 25.0 | 62.5 | 87.5 | 75.0 | 60.9 |
|  |  | HRD | 75.0 | 87.5 | 75.0 | 100.0 | 87.5 | 100.0 | 87.5 | 100.0 | 89.1 |
|  | 18 | SFT | 87.5 | 87.5 | 87.5 | 87.5 | 87.5 | 87.5 | 100.0 | 100.0 | 90.6 |
|  |  | HRD | 12.5 | 75.0 | 75.0 | 87.5 | 50.0 | 75.0 | 50.0 | 50.0 | 59.4 |
|  | 23 | SFT | 87.5 | 75.0 | 87.5 | 50.0 | 37.5 | 37.5 | 50.0 | 50.0 | 59.4 |
|  |  | HRD | 87.5 | 100.0 | 100.0 | 75.0 | 62.5 | 75.0 | 100.0 | 87.5 | 85.9 |
|  | 12 | SFT | 75.0 | 62.5 | 37.5 | 50.0 | 37.5 | 62.5 | 50.0 | 62.5 | 54.7 |
|  |  | HRD | 87.5 | 87.5 | 87.5 | 62.5 | 87.5 | 87.5 | 100.0 | 37.5 | 79.7 |
|  | 21 | SFT | 75.0 | 75.0 | 62.5 | 75.0 | 75.0 | 75.0 | 75.0 | 87.5 | 75.0 |
|  |  | HRD | 75.0 | 87.5 | 87.5 | 50.0 | 37.5 | 37.5 | 37.5 | 50.0 | 57.8 |
|  | 19 | SFT | 100.0 | 100.0 | 50.0 | 37.5 | 87.5 | 100.0 | 62.5 | 75.0 | 76.6 |
|  |  | HRD | 75.0 | 25.0 | 50.0 | 50.0 | 87.5 | 12.5 | 75.0 | 50.0 | 53.1 |
|  | 2 | SFT | 62.5 | 62.5 | 50.0 | 37.5 | 50.0 | 75.0 | 87.5 | 37.5 | 57.8 |
|  |  | HRD | 50.0 | 62.5 | 62.5 | 62.5 | 62.5 | 25.0 | 62.5 | 75.0 | 57.8 |
|  | 6 | SFT | 75.0 | 75.0 | 75.0 | 37.5 | 62.5 | 75.0 | 50.0 | 50.0 | 62.5 |
|  |  | HRD | 50.0 | 37.5 | 37.5 | 62.5 | 12.5 | 37.5 | 87.5 | 37.5 | 45.3 |
|  | 10 | SFT | 25.0 | 62.5 | 62.5 | 37.5 | 50.0 | 37.5 | 75.0 | 50.0 | 50.0 |
|  |  | HRD | 62.5 | 50.0 | 50.0 | 37.5 | 62.5 | 37.5 | 75.0 | 87.5 | 57.8 |
|  | 20 | SFT | 50.0 | 50.0 | 75.0 | 50.0 | 25.0 | 75.0 | 37.5 | 25.0 | 48.4 |
|  |  | HRD | 37.5 | 75.0 | 62.5 | 37.5 | 62.5 | 75.0 | 62.5 | 62.5 | 59.4 |
|  | 13 | SFT | 37.5 | 37.5 | 50.0 | 50.0 | 50.0 | 62.5 | 50.0 | 75.0 | 51.6 |
|  |  | HRD | 25.0 | 25.0 | 50.0 | 50.0 | 75.0 | 12.5 | 71.4 | 87.5 | 49.6 |
|  | 8 | SFT | 62.5 | 37.5 | 50.0 | 25.0 | 25.0 | 50.0 | 50.0 | 50.0 | 43.8 |
|  |  | HRD | 37.5 | 50.0 | 50.0 | 62.5 | 62.5 | 75.0 | 62.5 | 37.5 | 54.7 |
