# Supplementary material for: Cortical Regions Encoding Hardness Perception Modulated by Visual Information Identified by Functional Magnetic Resonance Imaging With Multivoxel Pattern Analysis
Source: Front Syst Neurosci. 2019 Oct 1;13:52. doi: 10.3389/fnsys.2019.00052 (PMC6779815; doi:10.3389/fnsys.2019.00052)
Supplement: Supplementary file 2 [file Table_2.docx]

**Supplementary material**

**Pilot study**

To examine whether visual modulation of hardness perception can be induced in the supine position, we conducted a pilot study before the main experiment. Seven healthy, right-handed participants (four males and three females, mean age: 21.7 ± 1.5 years, mean Edinburgh handedness score: 80.2 ± 22.7) were enrolled in the study. No participants overlapped with those in the main experiment. All apparatus, including the MRI scanner, polyurethane pads (soft, medium, and hard), and the platform with a mirror on the top, were the same as those used in the main experiment (see the Materials and Methods section of the main text). Participants underwent the hardness estimation task, while lying in the scanner. The procedure for the task was also the same as that of the main experiment, except for the triplet-based presentation of the specimens. Along with the three pad conditions (SFT, MED, and HRD), two timing conditions were employed: SYNC and ASYNC. Participants touched the pad with both hands synchronously (at the same time) or asynchronously (at alternate times) in conditions SYNC and ASYNC, respectively. They were required to touch the pad with both hands while observing the reflection of the left hand and report the subjective hardness of the pad perceived by the right (hidden) hand orally by magnitude estimation. In subject 1 – 6, each of the four conditions (SFT-SYNC, SFT-ASYNC, HRD-SYNC, and HRD-ASYNC) were presented four times in pseudorandomized order and either the two MED-SYNC and one MED-ASYNC or one MED-SYNC and two MED-ASYNC conditions were interleaved in a run (19 conditions/run). In subject 7, each of the four conditions (SFT-SYNC, SFT-ASYNC, HRD-SYNC, and HRD-ASYNC) were presented four times in pseudorandomized order and two MED-SYNC and two MED-ASYNC conditions were interleaved in a run (20 conditions/run). Subject 1 – 6 and 7 completed 10 and 8 runs, respectively. The perceived hardness was normalized individually by dividing by the maxima and averaged over all participants. Supplementary Figure S1 shows the results. The abscissa and ordinate indicate the pad conditions and the normalized perceived hardness, respectively. Two-way repeated measures ANOVA with main effects of TIMING (ASYNC vs. SYNC) and PAD (SFT vs. MED vs. HRD) revealed a significant main effect of PAD [*F_(2, 12)_* = 14.9, *p* < 0.001] as well as the interaction between them [*F_(2, 12)_* = 13.2, *p* < 0.001]. Post-hoc Ryan’s test revealed a significant difference in the perceived hardness between the conditions ASYNC and SYNC in conditions SFT [*F_(1, 18)_* = 10.7, *p* < 0.01] and HRD [*F_(1, 18)_* = 13.1, *p* < 0.01]. Moreover, there was a significant difference in the perceived hardness between conditions SFT and MED, HRD and MED, and SFT and HRD in condition SYNC (*p* < 0.01 for all comparisons), but not in ASYNC. Despite the small sample size, the result of the pilot study was consistent with that of our previous study (Katsuyama et al., 2018). Thus, we concluded that the visual modulation of the perception of hardness can be induced even in the supine position.

Reference

Katsuyama, N., Kikuchi-Tachi, E., Usui, N., Yoshizawa, H., Saito, A., and Taira, M. (2018). Effect of visual information on active touch during mirror visual feedback. Front Hum Neurosci 12, 424. doi: 10.3389/fnhum.2018.00424

**Supplementary Figure S1. Behavioral result of the pilot study.**

The perceived hardness was normalized individually to the maxima and averaged over all participants. The error bar indicates standard deviation. * indicates *p* < 0.01.
