# Supplementary figures and images for: Cortical Regions Encoding Hardness Perception Modulated by Visual Information Identified by Functional Magnetic Resonance Imaging With Multivoxel Pattern Analysis
Source: Front Syst Neurosci. 2019 Oct 1;13:52. doi: 10.3389/fnsys.2019.00052 (PMC6779815; doi:10.3389/fnsys.2019.00052)

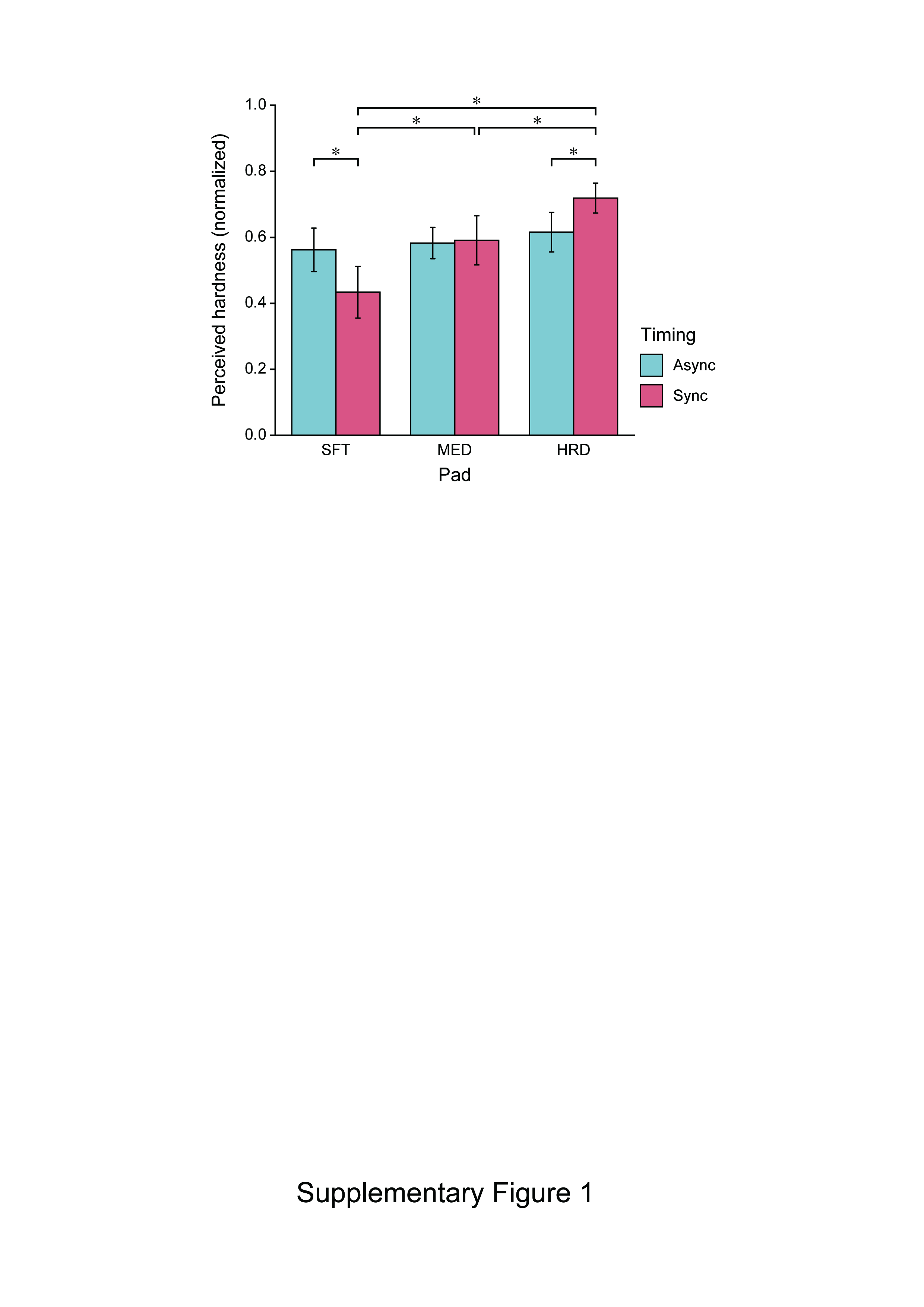

Supplement: Supplementary file 3 [file Image_1.tif]
